# Multiple renal cancer susceptibility polymorphisms modulate the HIF pathway

**DOI:** 10.1371/journal.pgen.1006872

**Published:** 2017-07-17

**Authors:** Steffen Grampp, Virginia Schmid, Rafik Salama, Victoria Lauer, Franziska Kranz, James L. Platt, James Smythies, Hani Choudhry, Margarete Goppelt-Struebe, Peter J. Ratcliffe, David R. Mole, Johannes Schödel

**Affiliations:** 1 Department of Nephrology and Hypertension, Universitätsklinikum Erlangen und Friedrich-Alexander-University Erlangen-Nürnberg, Erlangen, Germany; 2 NDM Research Building, University of Oxford, Old Road Campus, Headington, Oxford, United Kingdom; 3 Department of Computer Science 9, Friedrich-Alexander-Universität (FAU) Erlangen-Nürnberg, Erlangen, Germany; 4 Department of Biochemistry, Faculty of Science, Center of Innovation in Personalized Medicine, King Fahd Center for Medical Research, King Abdulaziz University, Jeddah, Saudi Arabia; National Cancer Institute (NCI), UNITED STATES

## Abstract

Un-physiological activation of hypoxia inducible factor (HIF) is an early event in most renal cell cancers (RCC) following inactivation of the von Hippel-Lindau tumor suppressor. Despite intense study, how this impinges on cancer development is incompletely understood. To test for the impact of genetic signals on this pathway, we aligned human RCC-susceptibility polymorphisms with genome-wide assays of HIF-binding and observed highly significant overlap. Allele-specific assays of HIF binding, chromatin conformation and gene expression together with eQTL analyses in human tumors were applied to mechanistic analysis of one such overlapping site at chromosome 12p12.1. This defined a novel stage-specific mechanism in which the risk polymorphism, rs12814794, directly creates a new HIF-binding site that mediates HIF-1α isoform specific upregulation of its target *BHLHE41*. The alignment of multiple sites in the HIF *cis*-acting apparatus with RCC-susceptibility polymorphisms strongly supports a causal model in which minor variation in this pathway exerts significant effects on RCC development.

## Author summary

Though numerous DNA polymorphisms that modulate the risk of developing cancer have been identified, understanding the functional role of extra-genic variation has so far proved difficult, in part because it is difficult to link such variation to a functional framework. To address this, we aligned extragenic renal cancer risk polymorphisms identified by genome-wide association studies, with DNA binding sites for the HIF transcription factors, which are constitutively activated in renal cancer and observed striking overlap. Detailed analysis of one overlapping polymorphism revealed that the risk allele creates a new HIF-binding site in a cell-type stage-specific enhancer of its target *BHLHE41*. Our study has provided new insights into renal specific oncogenesis, revealing multiple mechanisms by which modulation of specific aspects of HIF signalling impact on this form of cancer.

## Introduction

Successive advances in genetic analysis have provided insights into the biology of cancer. To date, such analyses have focused mainly on mutations that affect the integrity of transcribed genes. In contrast, it has so far proved difficult to interpret alterations in extragenic sequences, despite the existence of a great deal of data from large-scale genetic association and genome sequencing programs. Though recent studies suggest that the majority of extragenic sequences are functional, these functions are less well defined [1]. They are also likely to bear a more complex relationship to gene function than that of coding sequences. This problem is compounded by the extent of variation. Thus without a functional framework, it is difficult to impute causality by statistical methods that are agnostic to mechanism.

Nevertheless, progress has been made using genetic association studies that use commonly occurring polymorphisms in very large populations to define robust statistical associations with disease, including cancer. Should such statistically robust polymorphisms align to a common mechanistic framework then this might provide better insights into how extragenic polymorphism might operate in the development of cancer. Uncertainties in such an approach include difficulty in the definition of the target gene(s) on which extragenic polymorphism operates, and whether relevant functional pathways can be pre-defined.

In the current work we have sought to test this by alignment of renal cell cancer (RCC)-susceptibility polymorphisms, derived from published genome-wide association studies (GWAS) [2–6], with genomic binding sites for hypoxia-inducible factor (HIF), derived from chromatin immunoprecipitation (ChIP-seq) studies [7,8], and chromatin conformation analyses that identify distant targets of HIF-binding sites [9]. The von Hippel-Lindau tumor suppressor protein (pVHL) normally functions as a ubiquitin ligase effecting conditional destruction of HIF-α subunits in oxygenated cells [10,11]. Following inactivation of VHL in clear cell (cc)RCC, the HIF pathway is constitutively activated. Current evidence suggests that HIF activation contributes to the development of ccRCC [12–14], which accounts for approximately 85% of RCC, although effects of different components of the HIF pathway appear to be heterogeneous in their tumor promoting activities [15–18]. HIF pathway activation encompasses a large number of physiologically important activities that are dysregulated in ccRCC [19–21]. Therefore its activation in the kidney tubule, as an early event in the pathogenesis of ccRCC [12], provides a framework for testing for the extent of alignment between extragenic polymorphism influencing RCC-susceptibility and a pre-defined biological pathway that is relevant to that cancer.

Following the finding that RCC-susceptibility GWAS signals overlap HIF-binding sites at intergenic enhancers of *CCND1* and *MYC* [7,8], we undertook a systematic analysis of the overlap between published RCC GWAS signals and the HIF-transcriptional apparatus. Our data reveal additional associations between RCC-susceptibility polymorphisms and sites of HIF binding and demonstrate a highly significant overlap between these datasets. A comprehensive mechanistic analysis of one such site on chromosome (chr) 12p12.1 reveals a novel HIF-binding enhancer of the basic-helix-loop-helix transcription factor, BHLHE41, which is active in renal tubular cells. Allele-specific studies of this polymorphism in renal tubular cells, ccRCC cell lines, and human ccRCC material, suggests a novel stage-specific mechanism by which polymorphism tunes the output of the HIF pathway. Our findings reinforce the importance of the HIF pathway in ccRCC development and suggest that even minor modulation of this pathway is important.

## Results

### Germline variants associated with renal cancer preferentially affect the HIF pathway

We first examined whether genetic variants associated with an increased incidence of RCC preferentially affect the HIF transcriptional pathway by systematically examining for overlap with HIF-binding sites. A search of GWAS Central and PubMed databases identified 9 separate RCC-associated susceptibility loci (Table 1) that have reached genome-wide significance in at least one study [2–6]. For each locus, the single nucleotide polymorphism (SNP) that showed the most significant association with renal cancer was considered as the index SNP. Haplotype blocks associated with each RCC-associated locus were defined using 1000 Genomes CEU population data (all SNPs in high linkage disequilibrium (LD), r^2^ > 0.8, with each of the index SNPs). HIF-binding sites were defined by ChIP-seq analysis in two RCC cell lines (RCC4 and 786-O) and were present in a minimum of two out of 5 data sets (RCC4—HIF-1α, HIF-1β, HIF-2α and 786-O—HIF-1β, HIF-2α) [7,8].

**Table 1:** Published renal cancer-associated susceptibility loci.

Locus (chr)	Reported SNP	Reference
**2p21**	**rs7579899**	[2]
**12q24.31**	**rs4765623**	[2]
**11q13.3**	**rs7105934**	[2]
**12p12.1**	**rs718314**	[3]
**6q22.31**	**rs25422**	[3]
**8q24.21**	**rs6470588**	[4]
**2q22.3**	**rs12105918**	[5]
**5p13.3**	**rs10054504**	[5]
**1q24.1**	**rs3845536**	[6]

**Fig 1:**
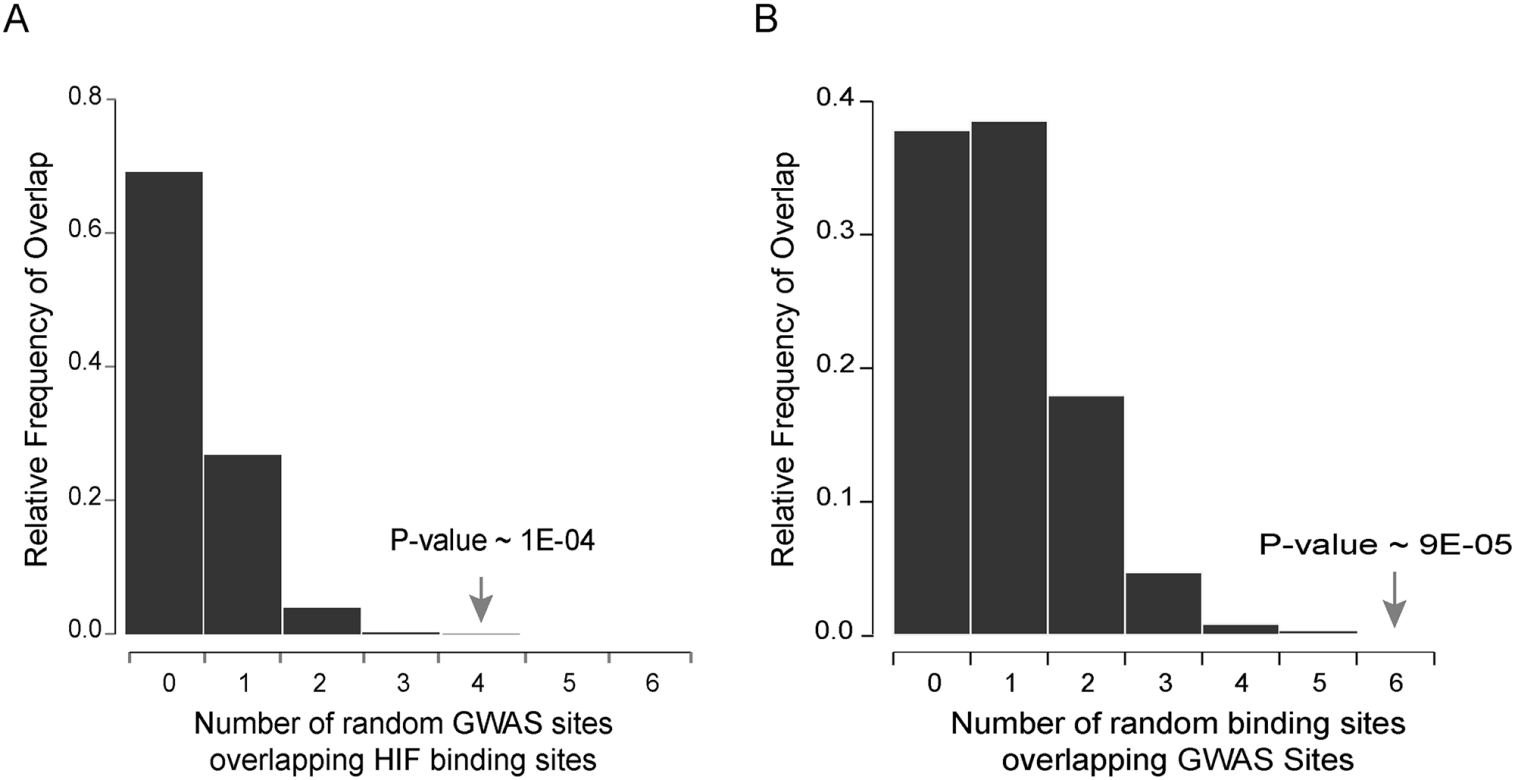
Non-random association between RCC GWAS loci and HIF-binding sites. 4 RCC GWAS loci overlapped with 6 HIF ChIP-seq peaks. **A)** To assess the significance of this overlap, the expected number of co-localization events was calculated by randomly shuffling the GWAS loci around the genome. This was repeated 100,000 times and the frequency distribution for the number of shuffled GWAS sites that overlapped a HIF-binding site was plotted. The probability of observing 4 or more GWAS loci overlapping a HIF ChIP-seq peak is 1x10^-4^ **B)** Conversely, HIF ChIP-seq peaks were randomly shuffled amongst all potential enhancer sites (as defined by the H3K27ac, H3K4me3, H3K4me1 in the same cell line 786-O), repeated 100,000 times and the frequency distribution for the number of shuffled HIF-binding sites that overlapped a GWAS site was plotted. The probability of observing 6 or more HIF-sites overlapping the GWAS sites is 9x10^-5^.

SNPs defining the haplotype blocks at 4 out of the 9 RCC-associated loci (chr 6q22.31, chr 8q24.21, chr 11q13.3, and chr 12p12.1) co-localized with a total of 6 HIF-binding sites. The number of GWAS loci co-localizing with HIF-binding sites strongly suggested a non-random association between the two sets of regions. To formally test this, the degree of overlap between the identified HIF ChIP-seq peaks and 9 randomly chosen regions (with a similar structure to the SNP structure within the RCC-associated haplotype blocks) was determined. The process was repeated 100,000 times to give a frequency distribution for the number of regions expected to overlap a HIF-binding site by random chance (Fig 1A). The observed overlap of 4 GWAS regions intersecting a HIF-binding site was highly significant with a random probability of occurrence of 1 x 10^−4^. Conversely, the GWAS regions were then fixed and the HIF-binding sites were randomly shuffled around all defined enhancers within the genome (as defined by H3K4me1, H3K4me3 and H3K27ac ChIP-seq in 786-O cells) and the frequency distribution after 100,000 iterations was plotted (Fig 1B). HIF-bound enhancers were significantly more likely to associate with the 9 RCC-associated GWAS loci than a comparable number of random enhancers (p = 9 x 10^−5^). Taken together, these analyses indicate that RCC-associated GWAS loci are preferentially affecting HIF-binding regions in RCC.

Both GWAS-identified disease-associated polymorphisms and HIF-binding sites are frequently intergenic. Consistent with this, three of the RCC-associated polymorphisms overlapped HIF-binding sites that are distant from any annotated gene promoter. Two, at chr 11q13.3 and chr 8q24.21, have been previously reported to affect long-range HIF-dependent expression of *CCND1* and *MYC* respectively [7,8]. We therefore focused on the chr 12p12.1 RCC-associated locus that overlapped a further promoter-distant HIF-binding site. Since this polymorphic HIF-binding site is intergenic, its transcriptional target was not immediately apparent.

### The RCC-associated polymorphic HIF-binding site at chr 12p12.1 regulates *BHLHE41* expression

**Fig 2:**
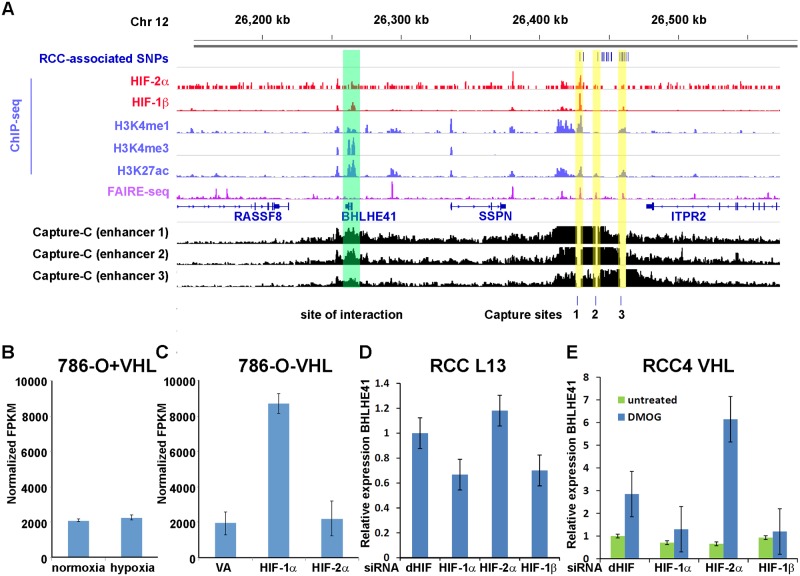
The chr 12p12.1 RCC risk locus encompasses a HIF-1-dependent enhancer that directly transactivates expression of *BHLHE41*. **A)** High-throughput sequencing analysis of the chr 12p12.1 locus in 786-O renal cancer cells—SNPs in high LD (r^2^>0.8) with the reported RCC-associated SNP (blue), ChIP-seq analysis of HIF-binding (red), ChIP-seq analysis of histone H3K4me1, H3K4me3 and H3K27ac modification (blue), FAIRE-seq analysis of chromatin accessibility (purple) and Capture-C analysis of chromatin interactions (black). SNPs in high LD with the reported RCC-associated SNP overlap with 3 enhancer sites as defined by chromatin accessibility and histone modification (highlighted in yellow) that interact with the distant *BHLHE41* promoter (highlighted in green), including a HIF-bound enhancer (Capture site 1). **B)** RNA-seq analysis of VHL-reconstituted 786-O renal cancer cells that express HIF-2α, but not HIF-1α does not show HIF-dependent regulation of BHLHE41. Values are mean FPKM (Fragments per kilobase of exon per million fragments mapped) from 3 independent RNA-seq experiments ± standard deviation. **C)** Overexpression of HIF-1α, but not HIF-2α induces BHLHE41 mRNA in RNA-seq analysis of 786-O cells (VA, vector alone). Values are mean FPKM values from 3 three independent RNA-seq experiments ± standard deviation. **D & E)** siRNA mediated suppression of HIF-1α and HIF-1β, but not HIF-2α in VHL-defective RCC L13 and VHL re-expressing RCC4 renal cancer cells (untreated or treated with 1 mM of the HIF-stabilizer DMOG) reduces expression of *BHLHE41* mRNA. qPCR values were normalized to the housekeeping gene HPRT and values from the untreated control siRNA (dHIF) sample. Values are mean relative expression from triplicate qPCR experiments ± standard deviation.

To determine the transcriptional target(s) of the HIF-binding site at the chr 12p12.1 locus, we first used a chromatin conformation assay (Capture-C, [9,22]) to examine for physical interaction between this region and distant gene promoters (Fig 2A). Oligonucleotides complementary to the DpnII restriction fragment containing the HIF-binding site were used to capture chromatin interactions in 786-O cells. Although not one of the two nearest genes flanking the HIF-binding site, significant interaction was observed between the polymorphic HIF-binding site at chr 12p12.1 (Capture site 1 in Fig 2A) and the promoter of the *BHLHE41* gene. Further SNPs in high LD with the index SNP rs718314 at chr 12p12.1 also overlapped two additional putative non-HIF-binding enhancers as defined by open chromatin and histone marks in RCC cells. Capture-C from each of these enhancers also showed interaction with the *BHLHE41* promoter (Fig 2A), indicating that each of the three enhancers within the polymorphic region associated with RCC may act on the *BHLHE41* promoter.

Having determined a physical association between the polymorphic HIF-binding site at the chr 12p12.1 locus and the *BHLHE41* promoter, we next examined for a functional effect of HIF on gene expression. Previous reports have described *BHLHE41* as a HIF-target gene in multiple cell lines, although hypoxic regulation has been attributed to promoter proximal binding of HIF [23]. However, in 786-O cells re-expressing VHL, we did not detect significant hypoxic regulation of *BHLHE41* (Fig 2B). This was despite strong HIF-2α binding at the chr 12p12.1 enhancer and physical interaction between this site and the *BHLHE41* promoter in these cells (Fig 2A). However, in keeping with a number of ccRCC cell lines, 786-O does not express functional HIF-1α [17]. In contrast, cell lines that do express HIF-1α show hypoxic regulation of *BHLHE41* [23]. This raised the possibility that *BHLHE41* is a specific transcriptional target of HIF-1α, but not HIF-2α. To test this hypothesis, we first examined *BHLHE41* gene expression in RNA-seq datasets in 786-O cells over-expressing exogenous HIF-1α or HIF-2α (Fig 2C)[17]. These showed clear induction of BHLHE41 mRNA levels by HIF-1α, but not HIF-2α overexpression. Similarly, suppression of HIF-1α, but not HIF-2α by siRNA reduced *BHLHE41* expression in in VHL-defective RCC L13 or VHL re-expressing RCC4 and VHL wild-type HK2 cells which were stimulated with a HIF prolyl hydroxylase inhibitor, dimethyloxalylglycine (DMOG)(Fig 2D and 2E, S1 Fig). This data indicates that, in renal cells, *BHLHE41* is a specific transcriptional target of HIF-1α, but not HIF-2α.

**Fig 3:**
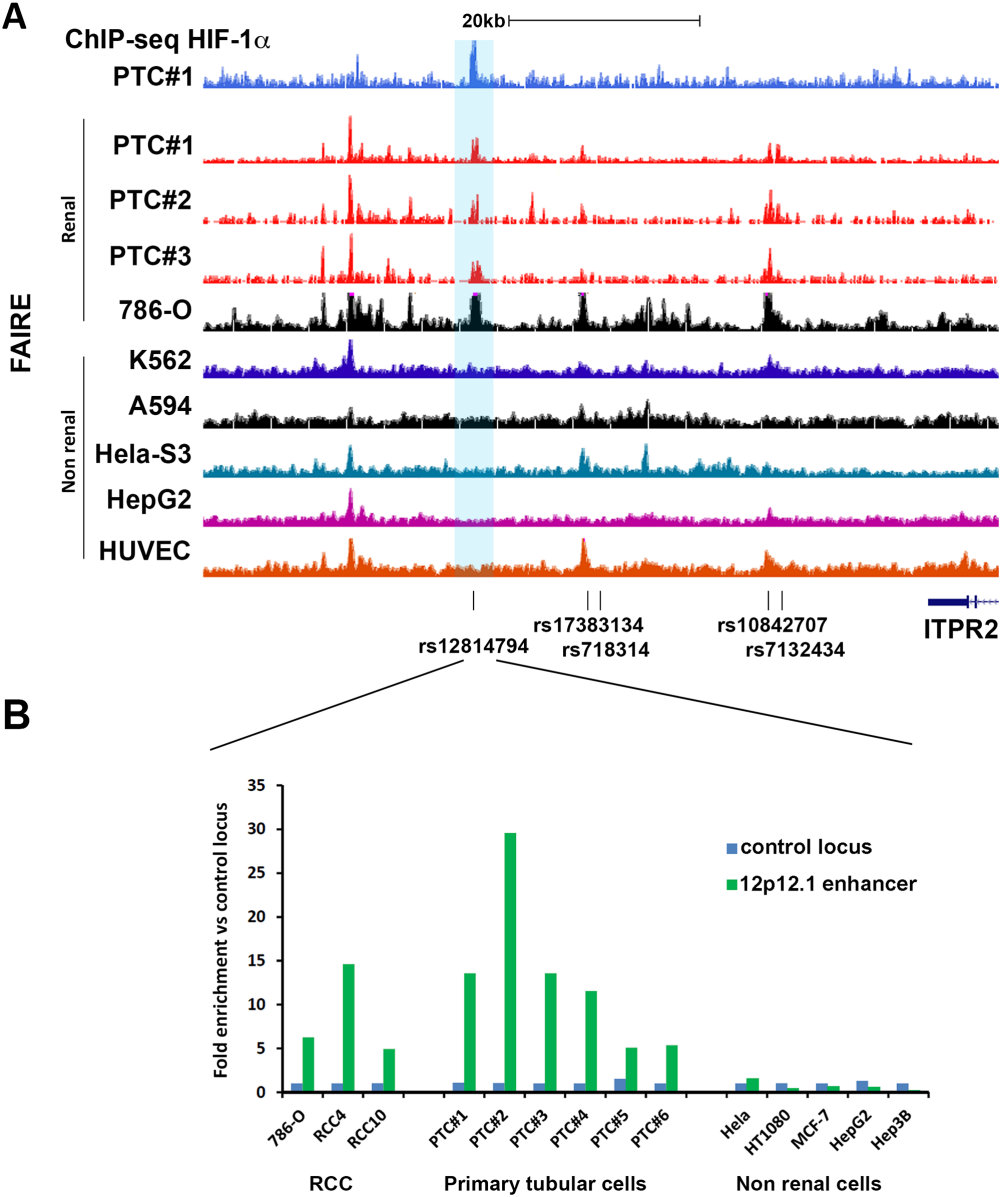
Open chromatin at the chr 12p12.1 RCC susceptibility locus overlaps with HIF-binding in primary renal tubular cells. **A)** HIF-1α ChIP-seq track for PTC isolated from individual #1 indicates binding of HIF at the chr 12p12.1 region in PTC (blue). HIF-1α was stabilized by 1 mM DMOG for 4h. FAIRE-seq data from primary renal tubular cells from three individuals (PTC#1–3, red) and ENCODE FAIRE-seq tracks of 786-O RCC and a selection of non-renal cells. Enhancer associated SNPs, which are in high LD with the tag SNP rs718314 are indicated below the tracks. **B)** FAIRE qPCR experiments confirm chromatin accessibility at the rs12814794 associated enhancer specifically in renal tubule derived cells (RCC and PTC) when compared to non-renal cells. Data is from one experiment per cell line. Average enrichment values compared to a putative non-enhancer region (control locus) are shown.

Although ccRCC commonly, and sometimes exclusively, expresses HIF-2α, it is derived from the renal tubular epithelium, which exclusively expresses HIF-1α and not HIF-2α [24]. We therefore sought to test whether primary renal tubular cells (PTC) manifest HIF-1α binding to this locus. Chromatin immunoprecipitation coupled to next generation DNA sequencing (ChIP-seq), or qPCR, revealed that this was indeed the case (Fig 3A, S2 Fig) with HIF-1α binding signals consistently observed in primary cultures of renal tubular epithelial cells. We next explored the renal specificity of the HIF-bound enhancer at the chr 12p12.1 locus, by performing formaldehyde-assisted isolation of regulatory elements (FAIRE) assays and also by reference to published data on chromatin accessibility from the ENCODE consortium. This revealed a strong bias towards open chromatin at this site in renal versus non-renal cell types (Fig 3A and 3B). Given that the other enhancers at the chr 12p12.1 RCC risk locus (Fig 2A) did not display this cell-type specificity in DNA accessibility, we focused further analysis on this site.

**Fig 4:**
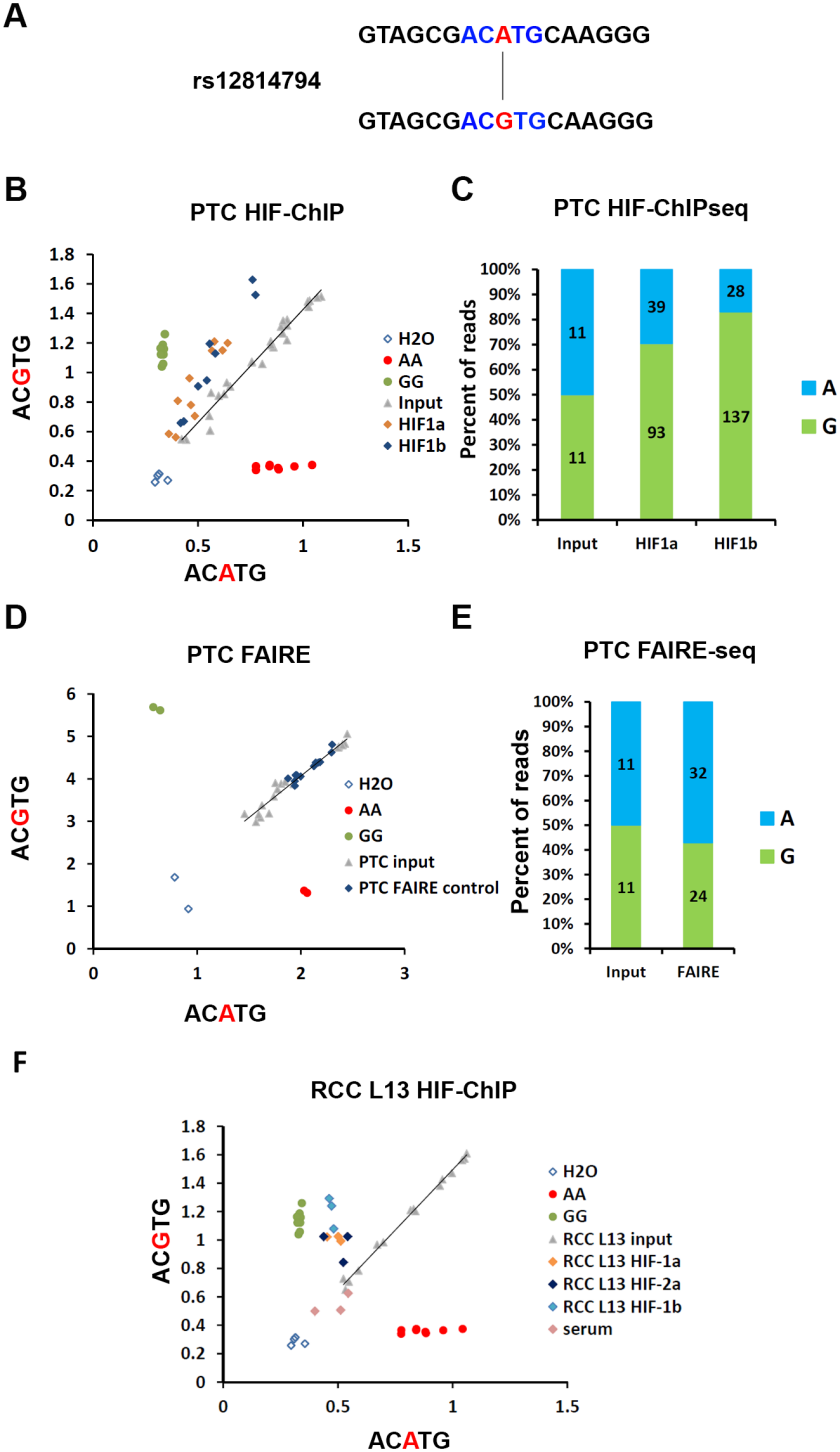
Allele specific HIF-binding at the rs12814794 associated enhancer. **A)** rs12814794 defines a one base pair exchange (A>G) which creates a HIF-binding motif (ACGTG). **B)** Allele-specific qPCR for rs12814794 from DNA fragments isolated in HIF-ChIP experiments (HIF-1α and HIF-1β) or input DNA from primary renal tubular cells (PTC, n = 4 individuals). DNA from individuals homozygous for the AA or GG genotype were used as positive controls. **C)** Quantification of the two different alleles (A or G) at rs12814794 in the ChIP-seq reads from HIF-1α and HIF-1β immunoprecipitations (PTC #1 Fig 3). **D)** Allele-specific qPCR for rs12814794 from DNA fragments isolated in FAIRE experiments or input DNA from primary renal tubular cells (PTC). DNA from individuals homozygous for the AA or GG genotype were used as positive controls. **E)** Quantification of the two different alleles (A or G) for rs12814794 present in reads from the FAIRE-seq experiment (see Fig 3a: FAIRE track for PTC#1) and sequencing of the input. **F)** Allele-specific qPCR for rs12814794 from HIF-ChIP (HIF-1α, HIF-2α and HIF-1β) experiments in RCC L13 cells. DNA from control serum immunoprecipitations or input DNA from RCC L13 was used as controls. DNA from homozygous individuals (AA or GG) were used as controls for the two genotypes.

Having determined both physical and functional associations between the HIF-binding site at the chr 12p12.1 RCC-susceptibility locus and expression of *BHLHE41*, we next examined for the effect of the RCC-associated polymorphism on HIF-binding and HIF-dependent gene expression. Inspection of the RCC-associated haplotype block revealed that the index SNP rs718314 is in high LD (r^2^ = 0.956) with rs12814794, which overlies the HIF ChIP-seq signal (Fig 3A). This variation is a one base pair A to G substitution, which directly disrupts or creates a hypoxia response element (HRE: RCGTG, R = A/G, disrupted HRE ACATG > intact HRE ACGTG) motif (Fig 4A). However the HIF ChIP-seq signal also coincides with a second non-polymorphic HRE motif within open chromatin that is potentially capable of binding HIF (S3 Fig). Both HIF-binding and (following re-expression) HIF-1α dependent *BHLHE41* gene expression, was observed in 786-O cells that were homozygous for the A allele at rs12814794 (Fig 2A and 2C), suggesting that this second HRE can be functional, at least in specific settings. Therefore, it was unclear to what extent the totality of HIF-binding to the chr 12p12.1 enhancer might be affected by the rs12814794 polymorphism that affects just one of the HREs.

To determine the effect of the rs12814794 polymorphism on HIF-binding and *BHLHE41* gene expression, we identified four individuals who are heterozygous for the rs12814794 SNP. Allele-specific ChIP-qPCR assays and ChIP-seq experiments in primary renal tubular cells derived from these individuals identified marked enrichment of the G allele (ACGTG—intact HRE) compared to the A allele in DNA fragments immunoprecipitated with antibodies against HIF-1α or its dimerization partner HIF-1β (Fig 4B and 4C). Taken together these results indicate that RCC-associated polymorphisms, which include those at rs12814794, affect binding of HIF at the chr 12p12.1 enhancer in primary renal tubular cells. Since previously defined RCC-associated polymorphisms that overlap HIF-binding sites appear to act indirectly by opening chromatin at these sites, we next tested for allele-specific effects on chromatin structure at this site by FAIRE and FAIRE-seq (Fig 4D and 4E). However in contrast with previously defined sites, we observed no allelic difference in chromatin accessibility.

We then examined for allele-specific HIF-binding in RCC cells using the VHL-defective RCC L13 cell line, which is heterozygous for rs12814794. ChIP-qPCR analysis using antibodies against HIF-1α, HIF-2α and HIF-1β again revealed preferential binding of HIF to the risk allele (Fig 4F). Thus, the RCC risk variant G at rs12814794 promotes HIF-binding to a renal-specific polymorphic HRE in both normal primary tubular cells and in renal cancer cells, likely due, at least in part, to the creation of a new core HRE (ACATG > ACGTG) within a region of open chromatin in renal cells.

**Fig 5:**
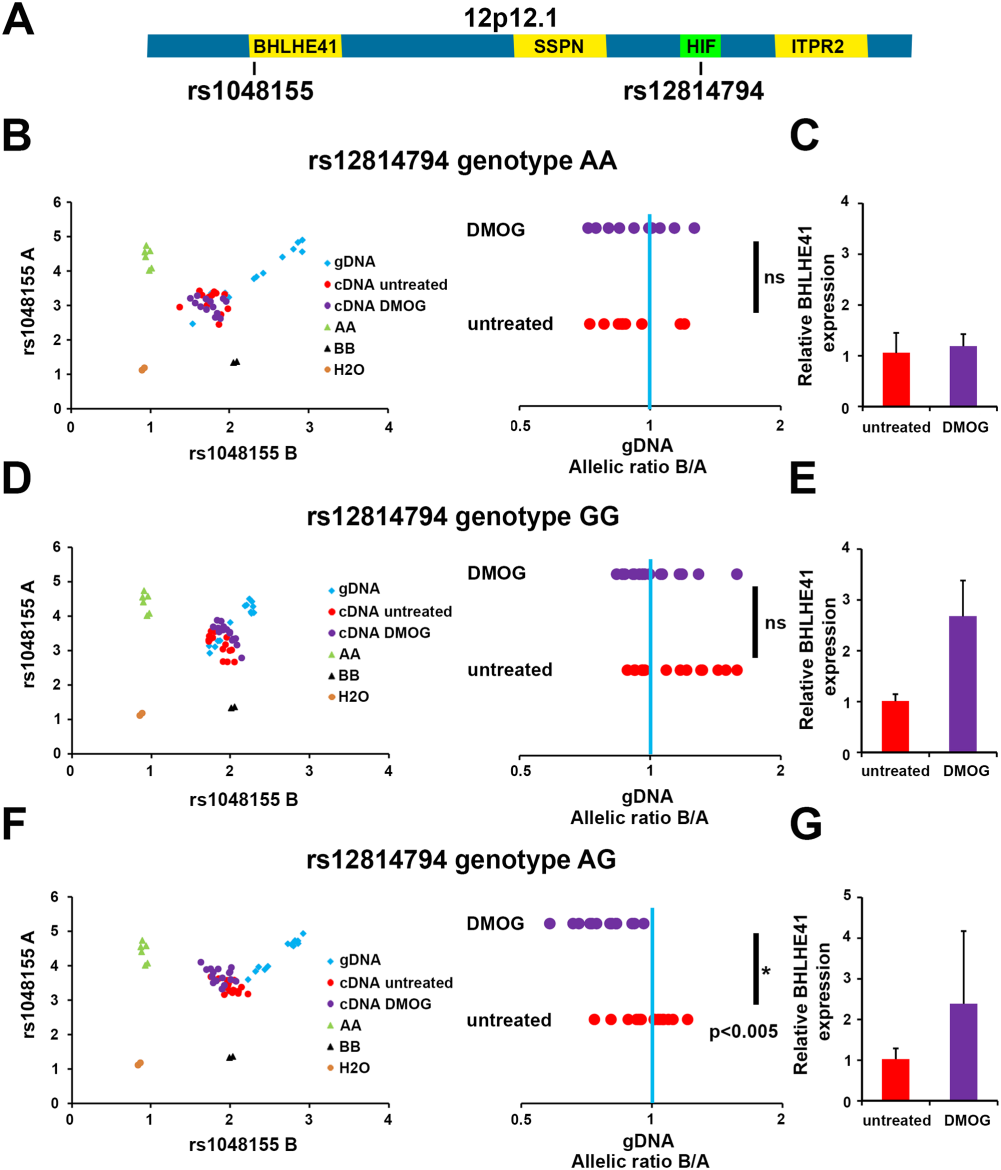
Allele-specific expression of *BHLHE41* is dependent on HIF-binding to rs12814794. **A)** Schematic view of the chr 12p12.1 locus. The SNP rs12814794 at the HIF-binding enhancer and the intragenic SNP rs1048155 in the 3’ UTR of *BHLHE41* are indicated. **B)** Allele-specific qPCR experiments for rs1048155 using genomic DNA (gDNA) or complementary DNA (cDNA) derived from primary tubular cells of an individual heterozygous for rs1048155 and homozygous (AA) for rs12814794. Cells were exposed to 1 mM DMOG or left untreated. The allelic ratios B/A for signals from untreated or DMOG treated cDNA normalized to gDNA are shown on the right. No difference in allelic expression is measured at the intragenic SNP (ns, non-significant). The blue bar indicates the allelic ratio of gDNA. **C)** In these cells *BHLHE41* expression is not induced by DMOG. Expression levels of *BHLHE41* were normalized to the housekeeping gene HPRT and to values from the untreated control. Expression qPCR was performed in technical quadruplicates on one biological sample. Values are mean ± standard deviation. **D)** Allele-specific qPCR using gDNA and cDNA from an individual homozygous for the G allele at the HIF-binding enhancer. No difference in allelic expression is measured at the intragenic SNP. **E)** *BHLHE41* expression is induced in these cells by 1 mM DMOG. Expression qPCR was performed in technical quadruplicates on one biological sample. Values are mean ± standard deviation. **F)** Allele specific qPCR for gDNA and cDNA using PTC from an individual heterozygous for both SNPs. A significant shift in the allelic ratio of the intragenic SNP rs1048155 is detectable in DMOG treated cells. Mann-Whitney-Wilcoxon test: p<0.005. **G)** *BHLHE41* expression is induced by 1 mM DMOG. Expression qPCR was performed in technical quadruplicates on one biological sample. Values are mean ± standard deviation.

Having shown that the RCC-associated polymorphism underlying the chr 12p12.1 HIF-binding site influences binding of HIF, we next tested for effects on expression of the transcriptional target, *BHLHE41*. We first identified individuals who were heterozygous for a SNP (rs1048155) in the 3’ UTR of the *BHLHE41* gene (Fig 5A). The phase of this SNP in relation to the RCC-associated SNP (rs12814794) is unknown, since these polymorphisms are in low LD (1000 Genomes, r^2^ = 0.107). However, it is possible to examine for the presence of allelic imbalance in the transcript, in individuals who are also heterozygous at the candidate RCC-associated SNP and contrast this with those who are homozygous at this locus.

Cells from individuals who were homozygous for either the A allele or the G allele at rs12814794 did not show any allelic imbalance in transcript abundance for the heterozygous rs104155 transcribed polymorphism (Fig 5B–5E, S4 and S5 Figs). Induction of HIF in these cells using the HIF prolyl hydroxylase inhibitor DMOG, also had no effect on allele-specific transcript abundance. Furthermore, cDNA from un-stimulated cells that were heterozygous for the RCC-associated polymorphism (rs12814794) did not show any allelic imbalance in expression of the rs104155 polymorphism either. However, when HIF was induced in these heterozygous cells using DMOG, preferential expression of one rs104155 allele over the other was observed (Fig 5F and 5G, S4 and S5 Figs).

**Fig 6:**
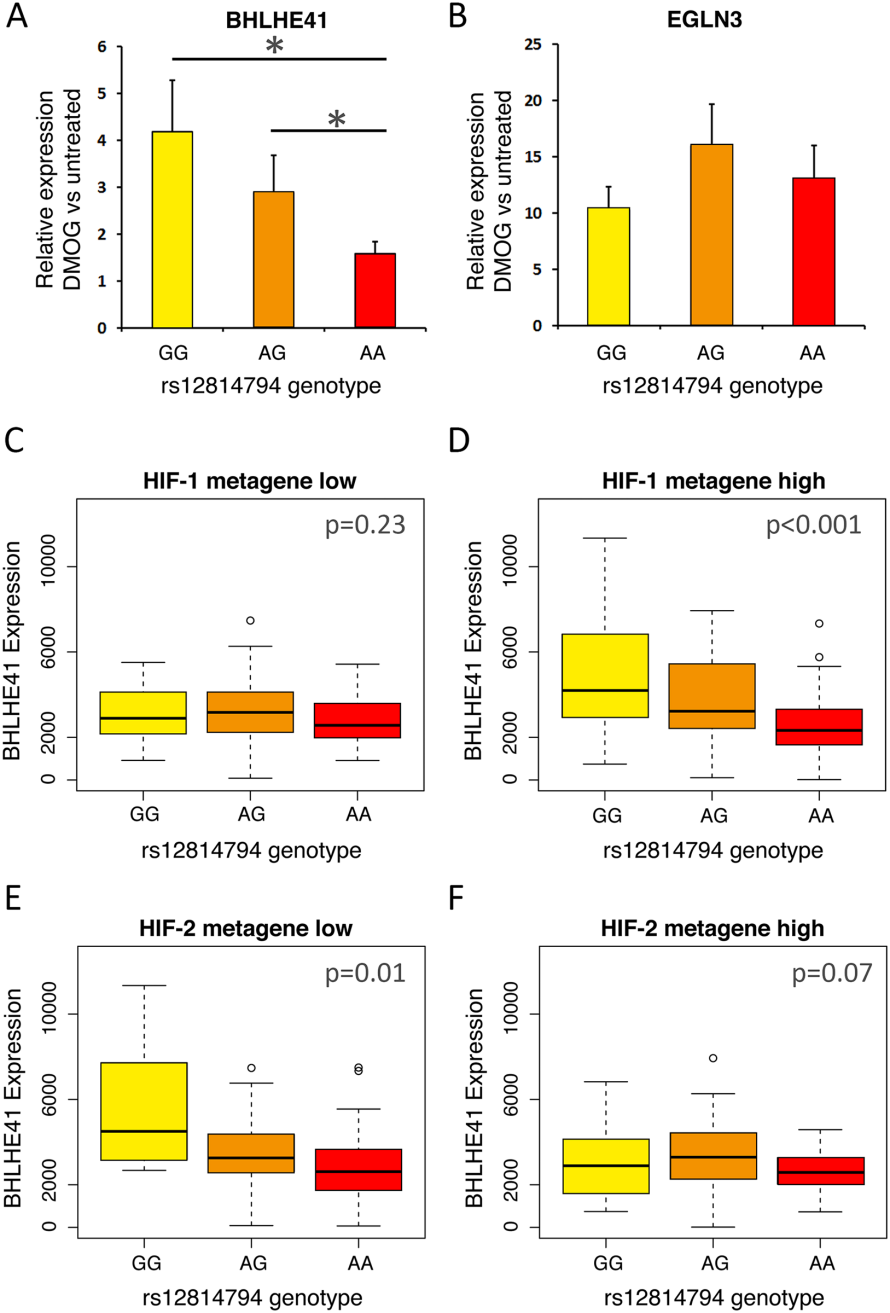
Interaction between HIF level, rs12814794 genotype and expression of *BHLHE41* mRNA. **A)** 33 genotyped primary tubular cell cultures (3 GG, 12 AG, and 18 AA) were exposed to 1 mM DMOG to induce HIF. *BHLHE41* mRNA expression was assayed by qPCR. The mean (± standard deviation) inducibility of *BHLHE41* mRNA compared to the respective untreated cells (= 1) was then plotted for each of the different rs12814794 genotypes. *, p<0.05—Student’s t-test. **B)** The mean inducibility of *EGLN3* mRNA levels in the same samples was plotted as control. **C-F)** Box-and-whisker plots showing the correlation between *BHLHE41* mRNA levels and genotype at rs12814794 in TCGA analysis of ccRCC tumors (KIRC). Tumors were first divided into tertiles according to expression of previously described HIF-1 or HIF-2 metagenes **C)** lower tertile for HIF-1 metagene expression, **D)** upper tertile for HIF-1 metagene expression, **E)** lower tertile for HIF-2 metagene expression, **F)** upper tertile for HIF-2 metagene expression. *χ*^2^-test was used to calculate p values for higher expression in GG individuals compared to AA individuals. Whiskers extend to 1.5 times of the inter quartile range.

To further explore the link between rs12814794 genotype and *BHLHE41* expression, we surveyed 33 primary tubular cell cultures for a correlation between the RCC-associated genotype and *BHLHE41* transcript level (Fig 6A). No difference in *BHLHE41* expression was observed between each of the three different rs12814794 genotypes under basal, non-stimulated conditions (S6 Fig). However, following HIF induction by DMOG, a significant increase in *BHLHE41* mRNA was observed in cells that carried at least one RCC-risk allele at the rs12814794 SNP, whilst the expression and induction of an control HIF target gene at an independent locus (*EGLN3*) was unaffected by the rs12814794 genotype (Fig 6A and 6B).

As has been previously reported [25], we observed an effect of RCC-associated polymorphisms (including rs12814794) at this locus on the expression of *BHLHE41* mRNA in ccRCC tumor samples analyzed by The Cancer Genome Atlas (TCGA). However, in our analysis of cell lines heterozygous for rs12814794, the effect of the polymorphism on gene expression required the presence of HIF-1α. Thus, we postulated that the association between the RCC-associated genotype and *BHLHE41* expression in the TCGA ccRCC analysis would be dependent on the activity of HIF-1α in those tumors. Since HIF-1α mRNA levels do not correlate well with HIF-1α functional activity, we constructed metagene arrays of genes that manifest strong transcriptional selectivity for either HIF-1α or HIF-2α [17]. We used these to stratify the TCGA ccRCCs according to low or high activity of HIF-1α or HIF-2α and re-examined for association between polymorphism at the chr 12p12.1 RCC-associated locus and the expression of *BHLHE41* (Fig 6C–6F). This analysis revealed that the chr 12p12.1 genotypic association with *BHLHE41* expression was conditional on high HIF-1α but not HIF-2α activity. Taken together, these analyses indicate that the RCC-associated polymorphism is capable of driving differential expression of *BHLHE41*, and that this action is at least in part dependent on the function of HIF-1α.

## Discussion

Our findings reveal highly significant overlap between human polymorphisms that modulate susceptibility to the development of RCC, as defined by genome-wide association studies, and *cis*-acting elements of the HIF transcriptional pathway, identified by HIF ChIP-seq analysis of RCC cell lines. These findings imply that extra-genic polymorphisms affecting the transcriptional output of the HIF pathway have an important bearing on the development of RCC. Nevertheless a caveat to this conclusion is that both the statistical definition of GWAS signals and imputation of function at polymorphic sites of HIF-binding are subject to uncertainty. At three loci, which coincide with intergenic enhancers of *CCND1* [7], *MYC* [8] and *BHLHE41* expression, we have performed detailed functional analyses that reveal mechanisms by which polymorphisms affect the expression of specific HIF target genes. At a fourth locus (chr 6q22.31) the overlapping HIF-binding site is close to the promoter of the gene encoding *CEP85L*. HIF-binding signals at this locus in 786–0 cells are less strong than at the other loci. Nevertheless data on transcript expression in clear cell renal carcinoma (TCGA) reveal upregulation in tumour versus normal kidney and association between the risk allele and increased expression of the transcript, suggesting a functional effect of the polymorphism (S7 Fig).

We focused our analysis of overlap with GWAS signals on DNA sequences that are immunoprecipitated with HIF to provide an objective, pan-genomic, measure of DNA sequences likely to influence the output of the HIF system. However, this definition is conservative, and will under-specify the totality of genomic sequences that modulate HIF pathway output. For instance, RCC-associated polymorphisms might operate on the HIF system in ‘*trans*’, as is suggested by one of RCC-associated GWAS signals that does not overlap the HIF ChIP-sequences, but lies within the *EPAS1* (HIF-2α) locus itself [2,26]. Furthermore, it is likely that the *cis*-acting apparatus at HIF-target genes operates over much larger distances than those identified by ChIP-seq analysis, which simply reflects the shearing of DNA on either side of sites physically bound to HIF. Thus, RCC-associated polymorphisms may influence expression of these genes in *cis*, either by altering local chromatin structure, or by affecting more distant regulatory sites that interact with the HIF-binding site. Such a possibility is suggested by the RCC-associated locus at chr 12q24.31, which lies within the first intron of the gene encoding the lipoprotein scavenger receptor class B member 1, *SCARB1*. Whilst the index RCC-associated GWAS SNP at this locus, is close (approximately 2 kb) to an adjacent HIF-ChIP-seq signal, SNPs in high linkage with the index SNP do not overlap it precisely [2,7] (S8 Fig). Nevertheless, this region has strong candidacy for modulation of *SCARB1* expression during RCC development. In keeping with this, we observed that *SCARB1* is a HIF transcriptional target in 786-O cells (S8A Fig), that its expression is upregulated in RCC tumor material when compared with surrounding normal kidney (S8B Fig), and that the risk polymorphism (at chr 12q24.31, [2]) is quantitatively associated with increased expression in ccRCC tumors in data from TCGA (S8C Fig). Taken together with the functional studies on specific loci in this and previous work, these findings suggest a remarkable coincidence between loci at which extra-genic human polymorphism influences risk of RCC, and loci at which such polymorphism modulates the output of the HIF pathway. A number of recent studies have drawn attention to the coincidence of GWAS signals with markers of functional elements in DNA [1,27,28]. However, to our knowledge, this is the clearest association to date of multiple defined genes in a single transcriptional pathway, with a particular type of cancer.

In keeping with most of the susceptibility determinants identified by GWAS, the effect of the risk allele appears to be small. Nevertheless, the work suggests that multiple polymorphisms with small quantitative effects on the output of the HIF system have effects on RCC that are clearly discernible at the level of clinical cancer development.

VHL-associated cancer is remarkably tissue-specific. Despite the general operation of the VHL-HIF system in the cellular response to hypoxia, VHL-associated cancer, which leads to constitutive up-regulation of HIF, is almost entirely confined to clear cell renal carcinoma, pheochromocytoma, and hemangioblastoma [10,29]. Interestingly, the HIF-binding sites that overlap RCC-susceptibility polymorphisms appear to manifest a strong bias towards activity in renal as opposed to non-renal cells [7,8]. Whether this reflects particularly important effects of these specific genes in the causation of ccRCC, is unclear. More generally, further work will be required to determine whether the repertoire of HIF target genes identified in these studies reflects an ‘oligogenic’ model for ccRCC-drive following HIF activation, or more simply, the limited ability of current GWAS to define such effects.

In addition to the general implications of multiple overlaps between RCC-susceptibility polymorphism and the HIF pathway, detailed analysis of the chr 12p21.1 locus has provided several new insights into RCC-susceptibility mechanisms. First, the work defines an intergenic polymorphism, which directly creates an HRE in intergenic open chromatin, in a region that is physically associated with a target gene promoter. This contrasts with previous RCC-susceptibility polymorphisms that do not affect HIF-binding directly at the core HRE, but appear to operate indirectly, at least in part by undefined mechanisms that alter the accessibility of the site within chromatin [7,8]. Second, our analyses strongly support a mechanism that is stage specific. Despite the binding of both HIF-α isoforms to the locus, our data indicates that, at least in the renal cells examined, *BHLHE41* target gene activation is tightly restricted to the HIF-1α isoform. The renal tubular epithelium, which is the presumed origin of ccRCC, exclusively expresses HIF-1α [24]. Studies of multiple pre-cancerous renal lesions in patients with familial VHL disease show a switch to HIF-2α expression in lesions with more neoplastic pathological features [12,30]; not infrequently HIF-2α is the only isoform expressed in advanced ccRCC, and in many ccRCC lines [11,16,18,31]. This suggests that this RCC-associated polymorphism acts early during ccRCC development and cannot operate by this mechanism once mature ccRCC has extinguished HIF-1α. That there is such pressure to modulate the output of HIF system at an early stage in ccRCC development is consistent first with evidence that VHL-inactivation itself (and hence general un-physiological up-regulation of HIF) is an early event in ccRCC development [12–14], and secondly with the existence of changes in HIF-α isoform expression associated with ccRCC development [16,18].

Although *BHLHE41* is transcriptionally regulated by HIF-1 but not HIF-2, we observe binding of both isoforms to the enhancer. Post-binding isoform specificity in transactivating activity has been described for many transcriptional targets of HIF [32,33]. In previous work, we have analyzed the chromatin interactions of HIF-binding sites and found many to be independent of the presence or absence of HIF [9]. Therefore, the observation that the enhancer-promoter interaction persists in a HIF-1 defective cell (786-O), whilst *BHLHE41* gene induction and the eQTL require HIF-1 is consistent with these findings.

During the course of this work, another report on the functional characterization of the chr 12p12.1 RCC-susceptibility locus was published [25]. Although *BHLHE41* is not the nearest gene to the susceptibility locus, this previous report also identified *BHLHE41* as the likely target gene, although a different mechanism of polymorphic function was proposed. Our direct demonstration of physical association between the enhancers overlapping the susceptibility locus, and the promoter of *BHLH41*, using chromatin conformation assays and our direct measurement of allele specific effects on the *BHLHE41* transcript in heterozygous cells strongly supports the assignment of *BHLHE41* as the target of the locus. The earlier report mainly used 786–0 cells, which do not express HIF-1α, for functional assays and did not identify the polymorphic function of the HIF-1α restricted enhancer that we reveal in the current work. Rather, a different region of the risk haplotype was proposed to operate through polymorphic binding of AP-1. In the current work, we took advantage of the inducible operation of HIF-1α in renal tubular cells to demonstrate that allele-specific expression of *BHLHE41* was conditional on both heterozygosity at the risk locus and on the induction of HIF-1α. This provided an unusual opportunity to distinguish the action of polymorphism at different regions of the risk haplotype. It clearly reveals that a specific polymorphism within that haplotype creates a new, functional, HIF-1α binding motif that is physically associated with and directly activates the *BHLHE41* target gene at the risk locus. These findings are, however, not mutually exclusive with the previous work implicating AP-1, and could indicate that the risk haplotype has more than one action, an early action dependent on HIF-1α and later effects mediated by AP-1.

A number of studies have indicated a role for BHLHE41 in tumor development, though in different settings, different effects have been observed. Thus, BHLHE41 has been shown to promote oncogenesis in renal and prostate cancer [25,34]. In contrast, in breast, gastric and pancreatic cancer cells, anti-tumorigenic effects have been observed [35–37]. It has also been reported that BHLHE41 acts directly on HIF to promote VHL-independent proteolysis and to reduce HIF-target gene expression [36]. A further possibility is that BHLHE41 could displace HIF at selected target gene loci. Interestingly, contrasting effects of HIF, particular HIF-1α, on tumorigenesis have also been observed in different contexts [38]. Thus, pro-tumorigenic effects of HIF-1α activation have been observed in breast cancer, consistent with the reported restriction of breast cancer growth by BHLHE41 through down-regulation of HIF-α [36]. In contrast, in ccRCC, anti-tumorigenic effects of the constitutive activation of HIF-1α have been observed [15,18]. An interesting possibility that is consistent with this is that in ccRCC the risk allele acts by increasing BHLHE41 expression and restricting anti-tumorigenic effects of HIF-1α at an early-stage in ccRCC development[15,18].

Irrespective of these effector mechanism(s), our data suggest that modest effects of human polymorphism on the HIF transcriptional output have important effects on the risk of VHL-associated ccRCC and that the HIF pathway is under strong selective pressure in this setting. More generally, the use of high-significance GWAS signals to define transcriptional pathways and targets that are subject to selective pressure during cancer development may provide a functional framework for identifying extragenic somatic mutation in similar regions as ‘mini-driver’ events [39].

## Materials and methods

### Cell culture

786-O, Hela, MCF-7, Hep3B, HT1080, and HepG2 were purchased directly from ATCC. RCC10 and RCC L13 cells were a gift from M. Wiesener; RCC4 cells were a gift from C.H. Buys. 786-O, Hela, MCF-7, Hep3B, HT1080, and HepG2 were purchased directly from ATCC. The identity of RCC4 and 786-O cells was confirmed through the presence in RNA-seq data sets of unique mutations in the coding region of the *VHL* gene (chr3: 10,183,725 C>G and chr3:10,183,841 G>del) that have been previously been described for each cell line. Cell lines were regularly tested for mycoplasma infection. Cell lines were grown in Dulbecco’s modified Eagle’s Medium, 100 U/ml penicillin, 100 μg/ml streptomycin and 10% fetal bovine serum (Sigma Aldrich). Healthy human kidney cortical tissue from patients undergoing tumor nephrectomy was used for tubular cell isolation as described previously [40,41]. Each patient gave written informed consent. The use of human tissue and the research conducted was approved by the local ethical committee at the University of Erlangen-Nürnberg (Ethik-Kommission der Medizinischen Faktultät der Friedrich-Alexander Universität Erlangen-Nürnberg; Erlangen, Germany). Approval numbers: 3755 and 173_15Bc. Samples were minced, digested with collagenase II (Gibco), and sieved through 100 μm and 70 μm filters. Primary human tubular cell cultures were maintained in Dulbecco’s modified Eagle’s medium/Ham’s F-12 supplemented with 2.5% (day 1) or 0% (day 2 onwards) fetal calf serum, 2 mM L-glutamine, 100 U/ml penicillin and 100 μg/ml streptomycin, 5 μg/ml insulin, 5 μg/ml transferrin, and 5ng/ml selenium (Sigma), triiodothyronine (T3, Sigma) 10 ng/ml, hydrocortisone 1 mg/ml, epidermal growth factor 100 μg/ml (Peprotech). Epithelial origin was confirmed by immunocytochemistry for N- and E-Cadherin. HT1080 cells were cultured in minimal essential medium 10% fetal calf serum, 2 mM L-glutamine, 100 U/ml penicillin and 100 μg/ml streptomycin. Sub-confluent cell cultures were exposed to 1 mM dimethyloxalylglycine (DMOG, Cayman) as indicated, to induce HIF. Previous analysis has shown striking concordance between the effects of DMOG and hypoxia on pangenomic patterns of both gene activation and HIF-binding [42,43]. We observed consistent effects of hypoxia, DMOG and direct intervention on HIF expression (both overexpression and RNAi) on *BHLHE41* mRNA levels. We therefore consider DMOG a good surrogate for hypoxia both in terms of HIF activation and more specifically in relation to HIF-dependent *BHLHE41* induction.

### Chromatin immunoprecipitation

Chromatin immunoprecipitation (ChIP) experiments were performed as previously described [7,43–45] using antibodies directed against HIF-1α (rabbit polyclonal, PM14 or Cayman Chemicals, Cay10006421), HIF-2α (rabbit polyclonal, PM9 or PM8), HIF-1β (rabbit polyclonal, Novus Biologicals, NB100-110), H3K4me1 (rabbit polyclonal, Millipore, #07–436), H3K4me3 (rabbit monoclonal, Cell Signaling Technology, #9751) or H3K27ac (rabbit polyclonal, Abcam, #ab4729). Non-immunized rabbit serum or purified normal rabbit IgG (Millipore, #12–370) were used as negative controls as appropriate.

### siRNA transfection and RNA isolation

siRNAs targeting HIF-1α and HIF-2α subunits and control siRNA against drosophila HIF have been previously described [42]. siRNA against ARNT/HIF-1β was purchased from Dharmacon (#L-007207-00-0005). siRNAs at a final concentration of 40 nM were transfected using Saint red (Synvolux) transfection reagent. Transfection was repeated after 24h and cells were harvested 48h after the initial transfection. RNA from cells or tissue was isolated using Tri Reagent (Sigma Aldrich) or peqGold total RNA kit (PeqLab) according to the manufacturer´s protocol and transcribed into cDNA using the high capacity cDNA reverse transcription kit (Life Technologies). qPCRs were performed on a StepOnePlus real-time PCR system (Applied Biosystems). For expression primers please see S1 Table.

### Formaldehyde-assisted isolation of regulatory elements (FAIRE)

FAIRE experiments were performed according to Giresi et al. [46,47], with the following modifications: Crosslinking was performed for 5 min at room temperature using 1% formaldehyde and DNA isolation was performed using three rounds of phenol chloroform extraction. SYBRgreen qPCR was performed on both FAIRE DNA and input DNA. Values were normalized to input DNA and compared to a non-enhancer control region. Primer sequences are listed in S1 Table.

### Capture-C assay

Experiments were performed as previously described [9,22]. Briefly, 3C libraries were generated from 786-O cells with DpnII and were sonicated to 200 bp. Indexed libraries were generated with NEBnext reagents (#E6000, # E7335, New England Biolabs). Capture enrichment was performed with the SeqCap EZ system (#06953212001, Roche/Nimblegen) following the manufacturer’s instructions. 1–2 μg of indexed library was incubated with 13 pmol of a pool of biotinylated oligos (Integrated DNA technologies or Sigma). A double capture protocol was followed with 48h and 24h hybridizations [9]. Capture efficiency was determined with qPCR relative to a standard curve of genomic DNA prior to sequencing.

### DNA extraction

DNA was isolated using DNA cell lysis buffer (NaCl 100 mM, TRIS pH 8.0 10 mM, EDTA 25 mM, SDS 0.5%, Proteinase K 0.1 mg/ml). After proteinase K (Roche) digest the isolation of DNA was performed with phenol chloroform and precipitated. DNA quality and concentration were measured by NanoDrop (Peqlab) and Qbit (Life Technologies), respectively.

### High throughput sequencing

ChIP-seq and FAIRE-seq library preparations were carried out using Illumina protocols and libraries were sequenced on HiSeq 2000/2500 platforms (Illumina). Sequences were mapped to NCBI build 37 (hg19) using BWA. Capture-C libraries were sequenced on the HiSeq 4000 (Illumina). Capture-C data was analyzed as previously described [9,22]. Briefly, reads were trimmed, *in silico* digested for DpnII and aligned to the GRCh37 (hg19) with Bowtie 1.0 and interaction frequencies were determined using CCanalyser2.pl. Significant interactions were called using a background model of distance dependent decay from the capture site [9].

### Accession codes

ChIP-seq data are available from the Gene Expression Omnibus: GSE67237 (HIF-2α and HIF-1β ChIP-seq in 786-O cells); GSE78113 (histone modifications in 786-O cells); GSM1011120 (FAIRE-seq in 786-O cells) and GSE101064 (FAIRE and ChIP-seq in human primary tubular cells).

### eQTL association and significance

TCGA level 3 RNA-Seq expression data for 450 clear cell Renal Cell Carcinoma patients was associated with Affymetrix Genome-Wide Human SNP Array 6.0 level 2 data on the same tumors, which included genotyping data for rs12814794. To identify significance of association between the SNP genotype and the expression of BHLHE41, RNA-Seq expression across the patients was fitted to a negative binomial Generalized Linear Model (GLM) against the genotype status. The likelihood ratio of this model versus a model that ignores genotype status was then computed. Finally, a Chi-Square test was used to call significance of the genotype coefficients in stratifying the patients. A previously published metagene set for high or low HIF signatures in the TCGA data was used to stratify the TCGA expression data [17].

### Allele-specific assays

To identify samples heterozygous for the common and rare alleles at rs1048155 and rs12814794 DNA from cell lines and primary tubular cells was genotyped using TaqMan assays (S1 Table, Life Technologies). For allele-specific assays, DNA from FAIRE and ChIP experiments as well as cDNA from primary renal tubular cell cultures was used. Genomic DNA from untreated samples from the same experiments was used in serial dilutions as an input control. Cell lines homozygous for each allele were used as positive controls. Data were analyzed using the TaqMan Genotyper Software V1.3 (Life Technologies). The mean ratio of minor allele / major allele (FAM/VIC) for the heterozygous input DNA derived from different dilutions was arbitrarily set to 1 and the ratios of DNA from assays (FAIRE; ChIP or cDNA) were normalized to the input DNA ratios. For the intragenic SNP rs1048155, allelic ratios of cDNA from DMOG treated primary tubular cells were compared to the allelic ratio of the corresponding untreated control cells.

### Data analysis

Statistical analyses for RNA expression were performed using a one-sample t-test comparing the mean with a hypothetical value of 1 or using a Mann-Whitney-Wilcoxon test if applicable using GraphPadPrism Version 5.00 (GraphPad Software Inc.).

### RNA-seq

RNA-seq analysis was performed on total RNA according to Illumina protocols using the HiSeq 2000 platform. 786-O cells re-expressing pVHL were exposed to normoxia or hypoxia (1%) for 16h. Data for experiments using 786-O cells overexpressing HIF-1α, HIF-2α or vector alone have been published previously (GSE67237) [17]. Illumina adaptor sequences were trimmed using Trimgalore (0.3.3). Reads were then aligned to GRCh37 using Tophat 2.0.8b (http://ccb.jhu.edu/software/tophat/index.shtml) and bowtie 1.0.0 (http://bowtie-bio.sourceforge.net/index.shtml) and non-uniquely mapping fragments excluded using SAMtools (0.1.19) [48]. Total read counts for each UCSC defined gene were extracted using HTSeq (0.5.4p3) with ‘intersection-strict’ mode. Fold-change and significance were determined using DESeq2 [49].

### Overlap of HIF-binding and RCC susceptibility

9 separate RCC-associated susceptibility loci were identified using a combined search of GWAS Central and PubMed databases [2–6]. For each locus, the SNP that showed the most significant association with renal cancer was considered as the index SNP. Haplotype blocks associated with each RCC-associated locus were defined using 1000 Genomes CEU population data (all SNPs in high LD, r^2^ > 0.8, with each of the index SNPs). HIF-binding sites were defined by ChIP-seq analysis in two RCC cell lines (RCC4 and 786-O) and were present in a minimum of two out of 5 data sets (RCC4—HIF-1α, HIF-1β, HIF-2α and 786-O—HIF-1β, HIF-2α). RCC-associated haplotype blocks and HIF-binding sites that overlap with each other were recorded. To determine the statistical significance of this overlap, we examined the intersection between the identified HIF ChIP-seq peaks and 9 randomly chosen regions (with a similar structure to the SNP structure within the RCC-associated haplotype blocks). The process was repeated 100,000 times to give a frequency distribution for the number of regions expected to overlap a HIF-binding site by random chance. The converse analysis was then performed by examining the overlap between the 9 GWAS-defined RCC-associated haplotype blocks and a set of sites (equivalent in number and width to the HIF-binding sites) that had been randomly selected from all defined enhancers within the genome (as defined by H3K4me1, H3K4me3 and H3K27ac ChIP-seq in 786-O cells). Again, the frequency distribution after 100,000 iterations was determined to define the probability of observing a given number of overlapping sites.

## Supporting information

S1 Fig *BHLHE41* is a HIF-1 target gene. Knock-down of HIF in HK-2 immortalized proximal tubular cells. Relative *BHLHE41* expression in cells treated with dHIF siRNA (control siRNA) or siRNA directed against HIF-1α, HIF-2α or HIF-1β. Expression levels are shown for untreated cells (green) or cells exposed to 1 mM DMOG (blue) for 16h to stabilize HIF protein. Values are mean ±SD normalized to the housekeeping gene HPRT and to values from control siRNA samples (dHIF) in untreated conditions. Triplicate PCR assays per condition from one knock-down experiment.
(PDF)

S2 Fig HIF-1 binds to the chr 12p12.1 locus in primary renal tubular cells (PTC). **A)** ChIP qPCR experiments at the chr 12p12.1 locus using DNA fragments isolated with antibodies against HIF-1α, HIF-2α or HIF-1β in cell lysates from 1 mM DMOG treated PTC. Values are mean ±SD percent of input DNA from 13 independent experiments. *, p<0.05 versus serum control. Student’s t-test. **B)** Relative mRNA levels for *BHLHE41* in cells from the same individuals as in a) exposed to 1 mM DMOG for 16h or left untreated. Values are mean ±SD. p<0.005 versus untreated control. One sample t-test.
(PDF)

S3 Fig Two HIF-binding motifs in the enhancer. **A)** FAIRE- and ChIP-seq signals at the chr 12p12.1 enhancer from primary renal tubular cells. The SNP rs12814794 is positioned in the centre of the HIF-binding signal. **B)** Extended sequence of the enhancer. Hypoxia response elements are marked in yellow.
(PDF)

S4 Fig Expression of a control HIF target gene in cells with different genotypes at rs12814794. Relative induction levels of the HIF target gene *EGLN3* located on chromosome 14 in primary renal tubular cells exposed to DMOG 1 mM for 16h (purple) or left untreated (red). Samples correspond to the same samples used in Fig 5. The genotype of the cells at rs12814794 is indicated at the top of each graph. Values are mean ± standard deviation from quadruplicate assays of one biological sample.
(PDF)

S5 Fig Allele-specific induction of *BHLHE41* expression in cells heterozygous for rs12814794. Allele-specific qPCR experiments for rs1048155 using genomic or complementary DNA derived from primary tubular cells of individuals **A)** homozygous AA or **B)** heterozygous AG for rs12814794 (independent individuals to Fig 5). Cells were exposed to 1 mM DMOG or left untreated. The allelic ratios B/A for rs1048155 are shown on the right. DMOG induces a significant shift in the allelic ratio in cells heterozygous for rs12814794 and with intact HRE. p<0.05 versus control. Mann-Whitney-Wilcoxon test.
(PDF)

S6 Fig Genotype-expression analysis in PTC. **A)** Expression levels of *BHLHE41* mRNA in primary renal tubular cells with different genotypes at rs12814794. Cells were exposed to DMOG 1 mM for 16h or left untreated. Values are normalized to the housekeeping gene *HPRT*. n = 18 for AA, n = 12 for AG, and n = 3 for GG. **B)** The expression levels of *EGLN3* mRNA are shown as a control.
(PDF)

S7 Fig The chr 6q22.31 RCC risk locus encompasses a HIF-binding site close to the promoter of *CEP85L*. **A)** High-throughput sequencing analysis of the chr 6q22.31 locus in 786-O renal cancer cells—SNPs in high LD (r^2^>0.8) with the reported RCC-associated SNP (blue), ChIP-seq analysis of HIF binding (red), ChIP-seq analysis of histone H3K4me1, H3K4me3 and H3K27ac modification (blue), FAIRE-seq analysis of chromatin accessibility (purple) and RNA-seq analysis (green). SNPs in high LD with the reported RCC-associated SNP overlap a HIF ChIP-seq signal. **B)** RNA-seq analysis of 72 paired ccRCC and surrounding kidney samples in TCGA database showing expression of *CEP85L* mRNA. **C)** Box-and-whisker plots showing the correlation between *CEP85L* mRNA levels and genotype at rs9481825 (the genotyped SNP in highest LD with the index SNP, rs25422 in TCGA analysis of ccRCC tumors).
(PDF)

S8 Fig The chr 12q24.31 RCC risk locus lies adjacent to a HIF-2-dependent enhancer that directly transactivates expression of *SCARB1*. **A)** High-throughput sequencing analysis of the chr 12q24.31 locus in 786-O renal cancer cells—SNPs in high LD (r^2^>0.8) with the reported RCC-associated SNP (blue), ChIP-seq analysis of HIF binding (red), ChIP-seq analysis of histone H3K4me1, H3K4me3 and H3K27ac modification (blue), FAIRE-seq analysis of chromatin accessibility (purple) and RNA-seq analysis (green). SNPs in high LD with the reported RCC-associated SNP lie close to, but do not overlap a HIF ChIP-seq signal in the *SCARB1* gene. **B)** RNA-seq analysis of 72 paired ccRCC and surrounding kidney samples in TCGA database showing increased levels of *SCARB1* in ccRCC. **C)** Box-and-whisker plots showing the correlation between *SCARB1* mRNA levels and genotype at rs10846753 (the genotyped SNP in highest LD with the index SNP, rs4765623 in TCGA analysis of ccRCC tumors).
(PDF)

S1 Table Primers and PCR assays. (PDF)
