# Supplementary figures and images for: Multiple renal cancer susceptibility polymorphisms modulate the HIF pathway
Source: PLoS Genet. 2017 Jul 17;13(7):e1006872. doi: 10.1371/journal.pgen.1006872 (PMC5536434; doi:10.1371/journal.pgen.1006872)

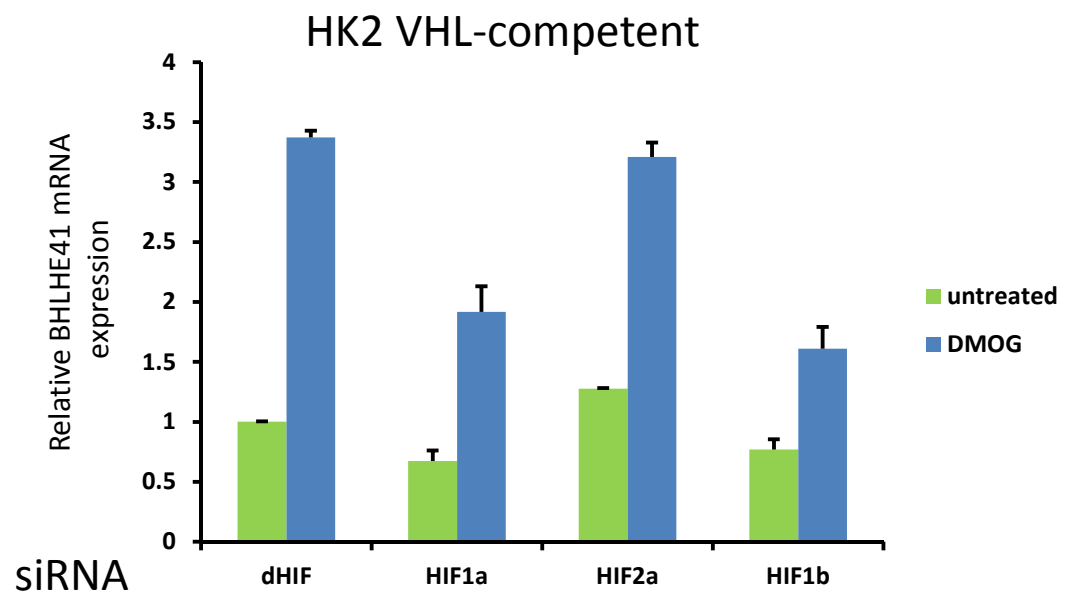

Supplement: S1 Fig — Knock-down of HIF in HK-2 immortalized proximal tubular cells. Relative BHLHE41 expression in cells treated with dHIF siRNA (control siRNA) or siRNA directed against HIF-1α, HIF-2α or HIF-1β. Expression levels are shown for untreated cells (green) or cells exposed to 1 mM DMOG (blue) for 16h to stabilize HIF protein. Values are mean ±SD normalized to the housekeeping gene HPRT and to values from control siRNA samples (dHIF) in untreated conditions. Triplicate PCR assays per condition from one knock-down experiment. (PDF) [file pgen.1006872.s001.pdf]

**A**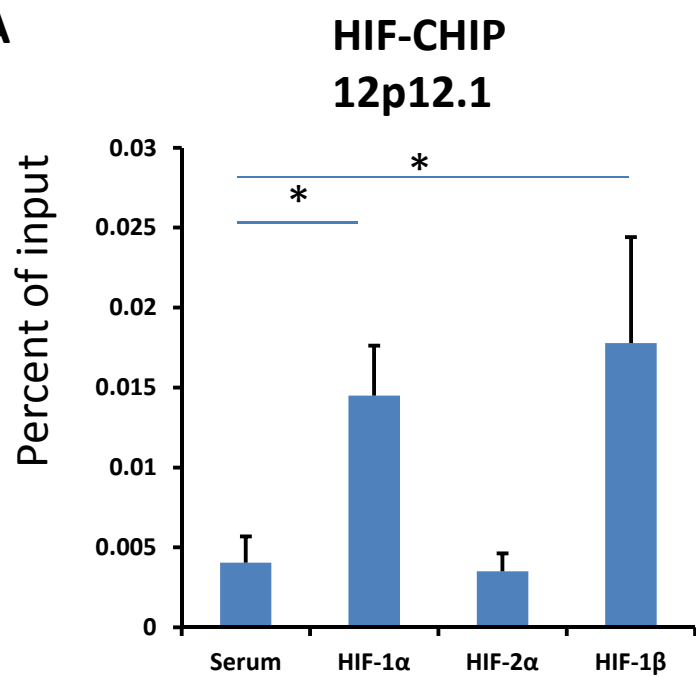**B**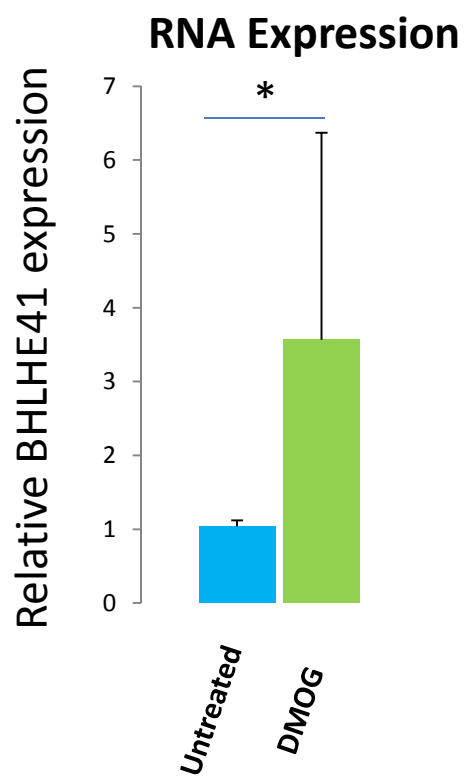

Supplement: S2 Fig — A) ChIP qPCR experiments at the chr 12p12.1 locus using DNA fragments isolated with antibodies against HIF-1α, HIF-2α or HIF-1β in cell lysates from 1 mM DMOG treated PTC. Values are mean ±SD percent of input DNA from 13 independent experiments. *, p<0.05 versus serum control. Student’s t-test. B) Relative mRNA levels for BHLHE41 in cells from the same individuals as in a) exposed to 1 mM DMOG for 16h or left untreated. Values are mean ±SD. p<0.005 versus untreated control. One sample t-test. (PDF) [file pgen.1006872.s002.pdf]

*EGLN3* expression

rs12814794 genotype

AA

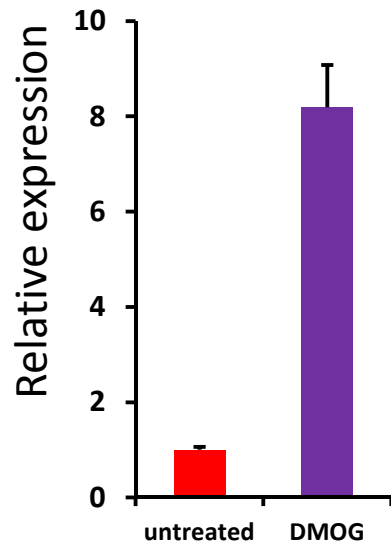

GG

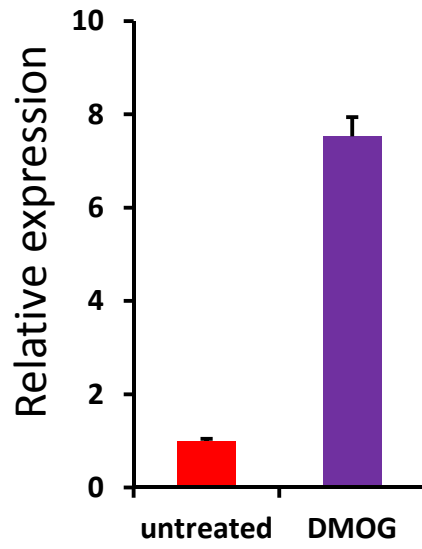

AG

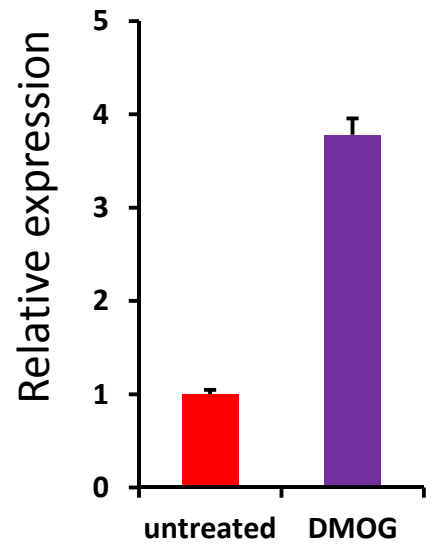

Supplement: S4 Fig — Relative induction levels of the HIF target gene EGLN3 located on chromosome 14 in primary renal tubular cells exposed to DMOG 1 mM for 16h (purple) or left untreated (red). Samples correspond to the same samples used in Fig 5. The genotype of the cells at rs12814794 is indicated at the top of each graph. Values are mean ± standard deviation from quadruplicate assays of one biological sample. (PDF) [file pgen.1006872.s004.pdf]

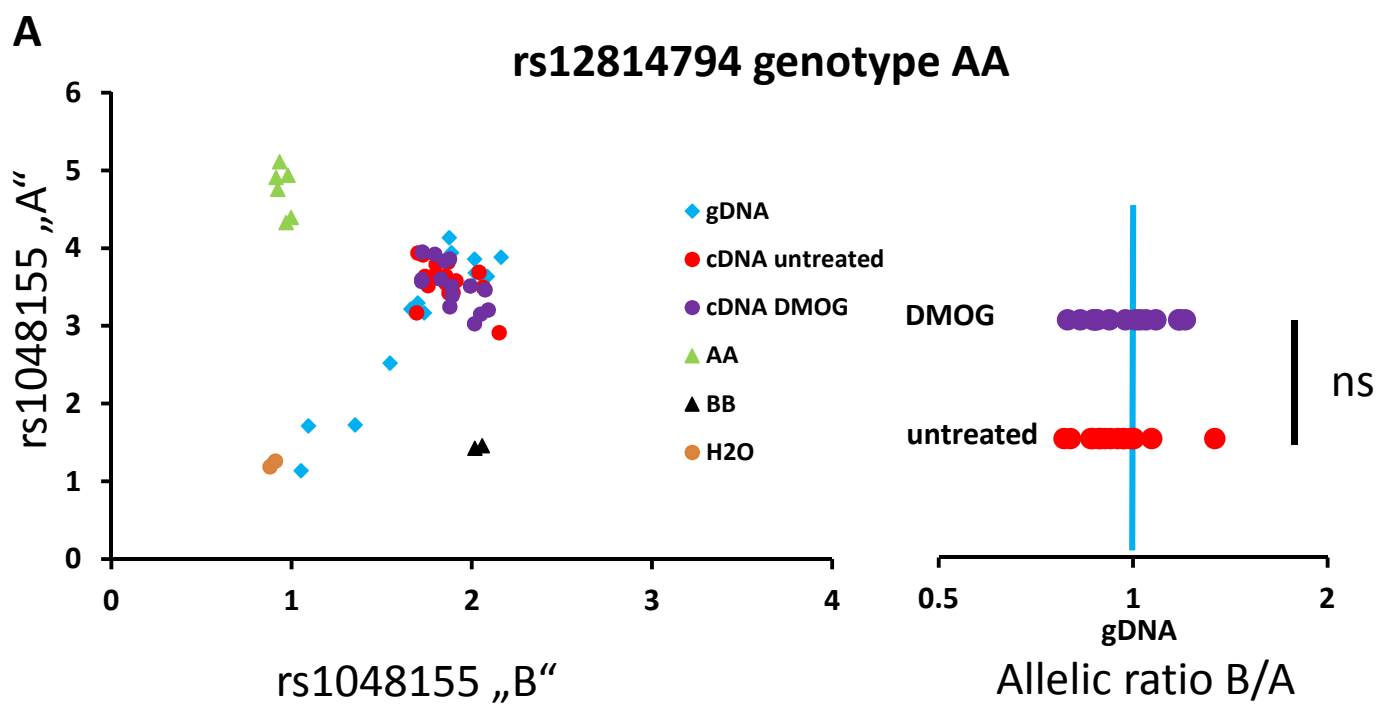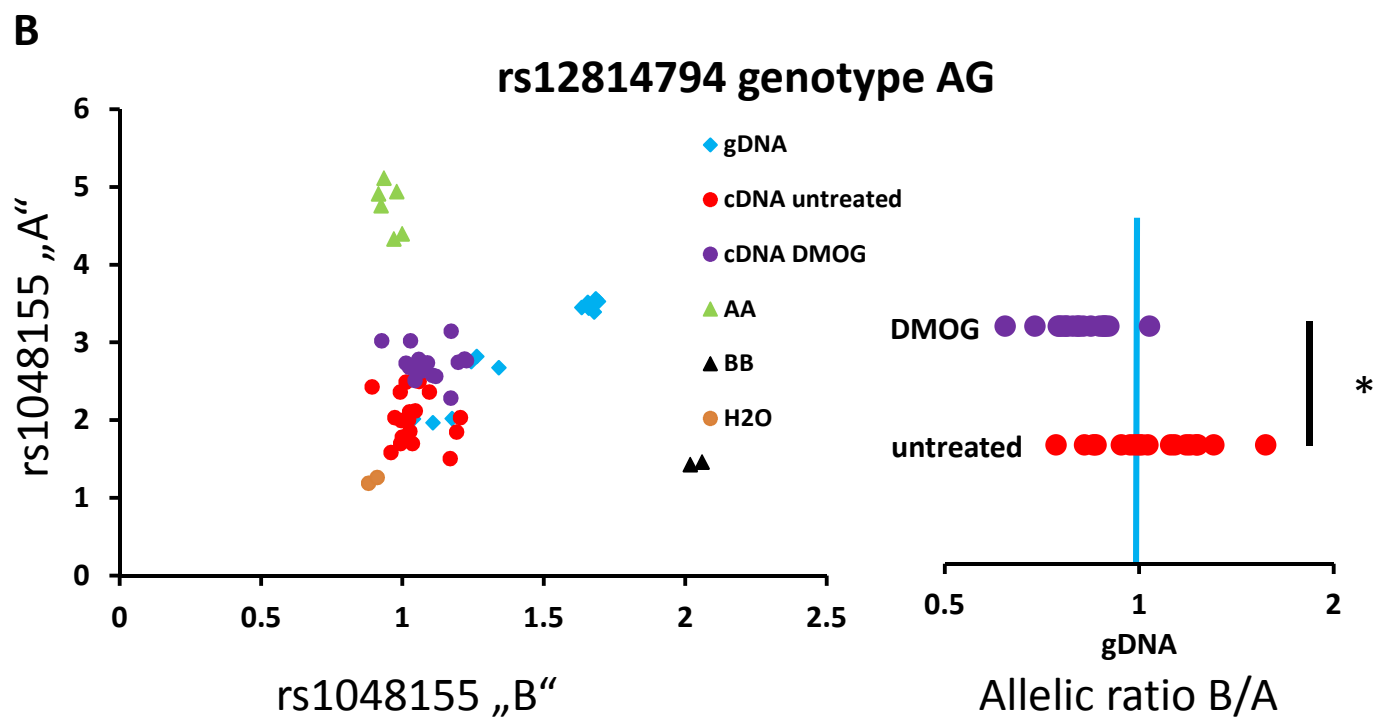

Supplement: S5 Fig — Allele-specific qPCR experiments for rs1048155 using genomic or complementary DNA derived from primary tubular cells of individuals A) homozygous AA or B) heterozygous AG for rs12814794 (independent individuals to Fig 5). Cells were exposed to 1 mM DMOG or left untreated. The allelic ratios B/A for rs1048155 are shown on the right. DMOG induces a significant shift in the allelic ratio in cells heterozygous for rs12814794 and with intact HRE. p<0.05 versus control. Mann-Whitney-Wilcoxon test. (PDF) [file pgen.1006872.s005.pdf]

**A**

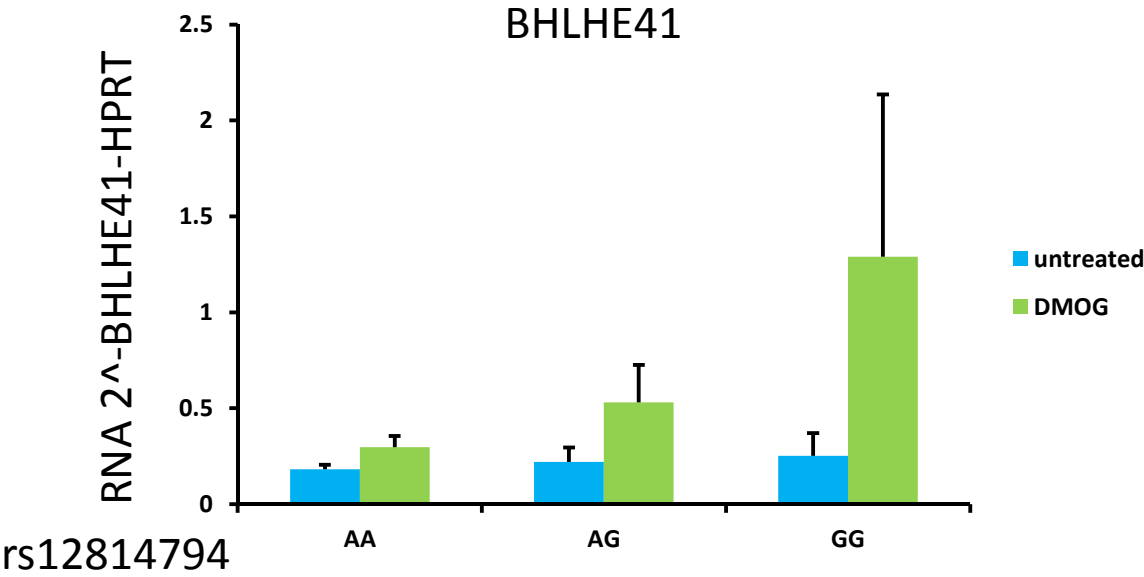

**B**

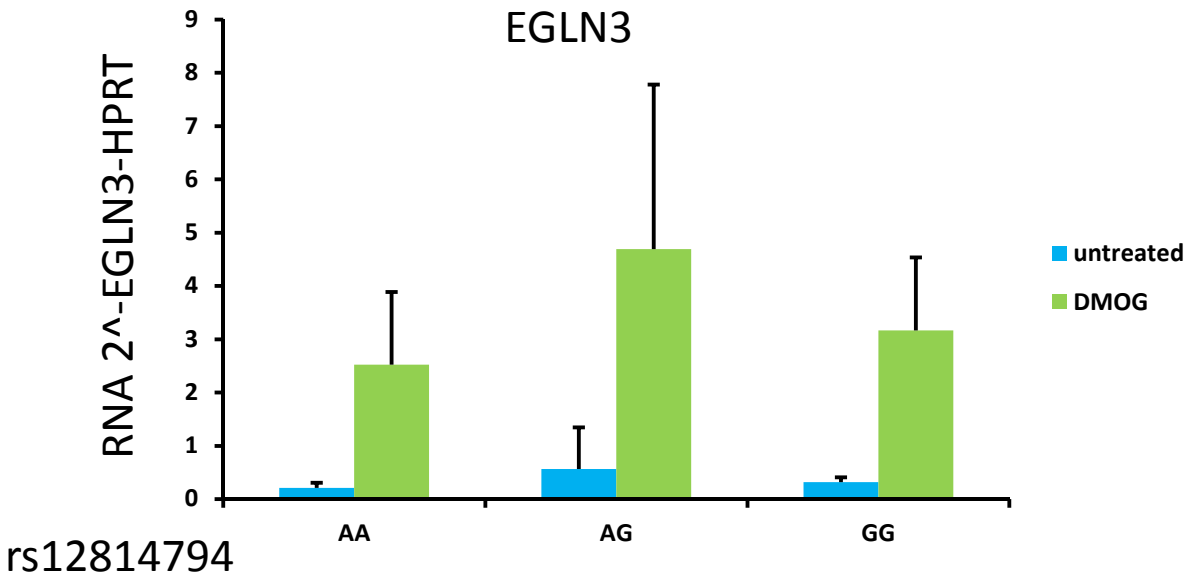

Supplement: S6 Fig — A) Expression levels of BHLHE41 mRNA in primary renal tubular cells with different genotypes at rs12814794. Cells were exposed to DMOG 1 mM for 16h or left untreated. Values are normalized to the housekeeping gene HPRT. n = 18 for AA, n = 12 for AG, and n = 3 for GG. B) The expression levels of EGLN3 mRNA are shown as a control. (PDF) [file pgen.1006872.s006.pdf]

**A**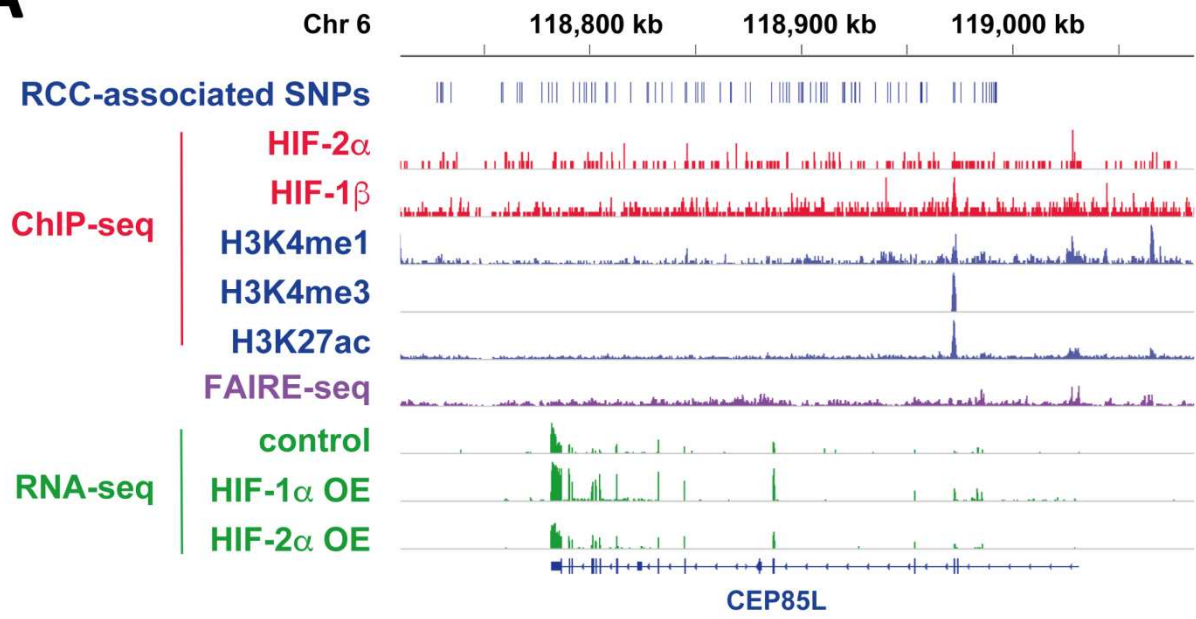**B**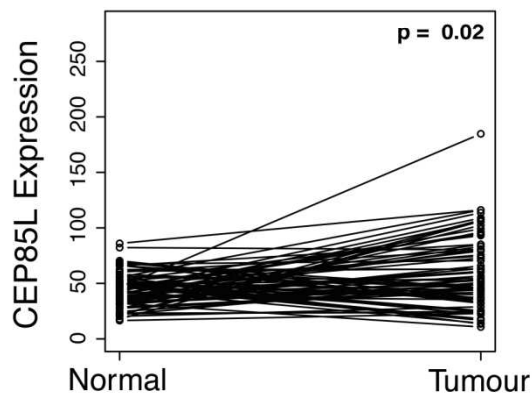**C**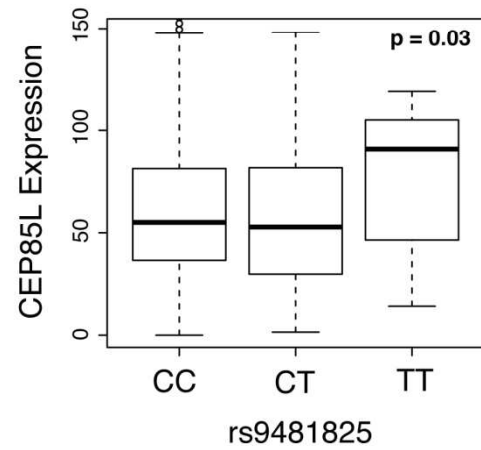

Supplement: S7 Fig — A) High-throughput sequencing analysis of the chr 6q22.31 locus in 786-O renal cancer cells—SNPs in high LD (r2>0.8) with the reported RCC-associated SNP (blue), ChIP-seq analysis of HIF binding (red), ChIP-seq analysis of histone H3K4me1, H3K4me3 and H3K27ac modification (blue), FAIRE-seq analysis of chromatin accessibility (purple) and RNA-seq analysis (green). SNPs in high LD with the reported RCC-associated SNP overlap a HIF ChIP-seq signal. B) RNA-seq analysis of 72 paired ccRCC and surrounding kidney samples in TCGA database showing expression of CEP85L mRNA. C) Box-and-whisker plots showing the correlation between CEP85L mRNA levels and genotype at rs9481825 (the genotyped SNP in highest LD with the index SNP, rs25422 in TCGA analysis of ccRCC tumors). (PDF) [file pgen.1006872.s007.pdf]

**A**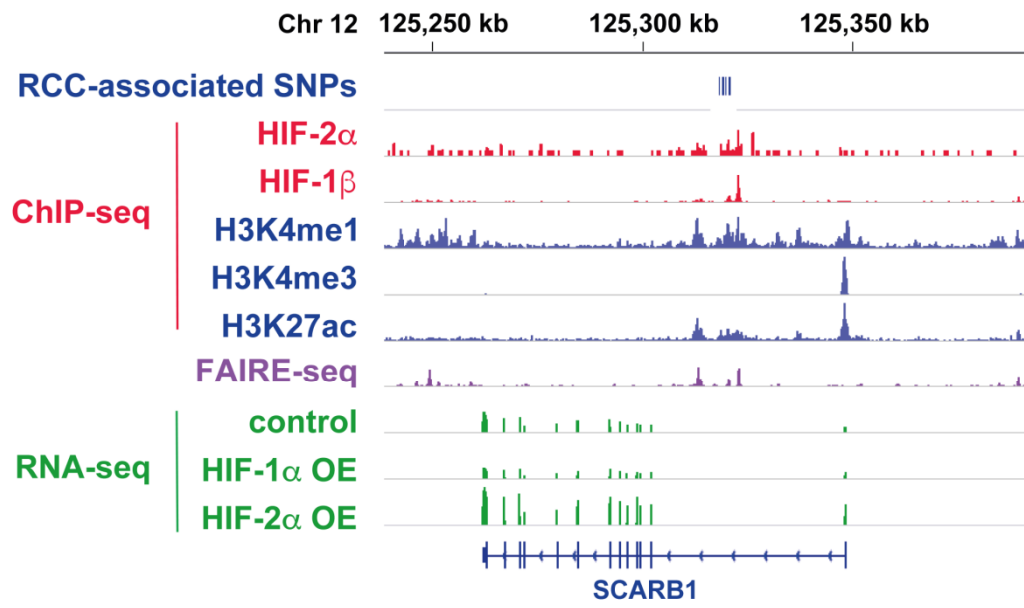**B**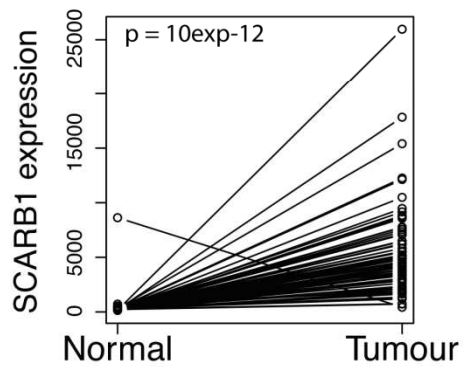**C**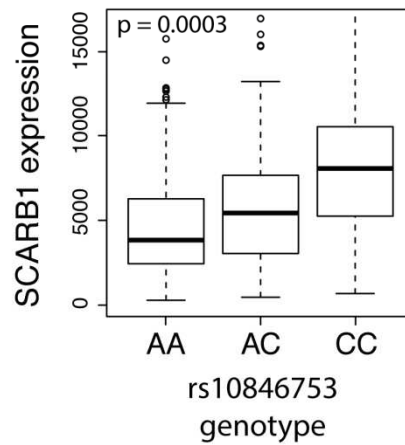

Supplement: S8 Fig — A) High-throughput sequencing analysis of the chr 12q24.31 locus in 786-O renal cancer cells—SNPs in high LD (r2>0.8) with the reported RCC-associated SNP (blue), ChIP-seq analysis of HIF binding (red), ChIP-seq analysis of histone H3K4me1, H3K4me3 and H3K27ac modification (blue), FAIRE-seq analysis of chromatin accessibility (purple) and RNA-seq analysis (green). SNPs in high LD with the reported RCC-associated SNP lie close to, but do not overlap a HIF ChIP-seq signal in the SCARB1 gene. B) RNA-seq analysis of 72 paired ccRCC and surrounding kidney samples in TCGA database showing increased levels of SCARB1 in ccRCC. C) Box-and-whisker plots showing the correlation between SCARB1 mRNA levels and genotype at rs10846753 (the genotyped SNP in highest LD with the index SNP, rs4765623 in TCGA analysis of ccRCC tumors). (PDF) [file pgen.1006872.s008.pdf]
