# Supplementary material for: Multiple renal cancer susceptibility polymorphisms modulate the HIF pathway
Source: PLoS Genet. 2017 Jul 17;13(7):e1006872. doi: 10.1371/journal.pgen.1006872 (PMC5536434; doi:10.1371/journal.pgen.1006872)
Supplement: S3 Fig — A) FAIRE- and ChIP-seq signals at the chr 12p12.1 enhancer from primary renal tubular cells. The SNP rs12814794 is positioned in the centre of the HIF-binding signal. B) Extended sequence of the enhancer. Hypoxia response elements are marked in yellow. (PDF) [file pgen.1006872.s003.pdf]

A

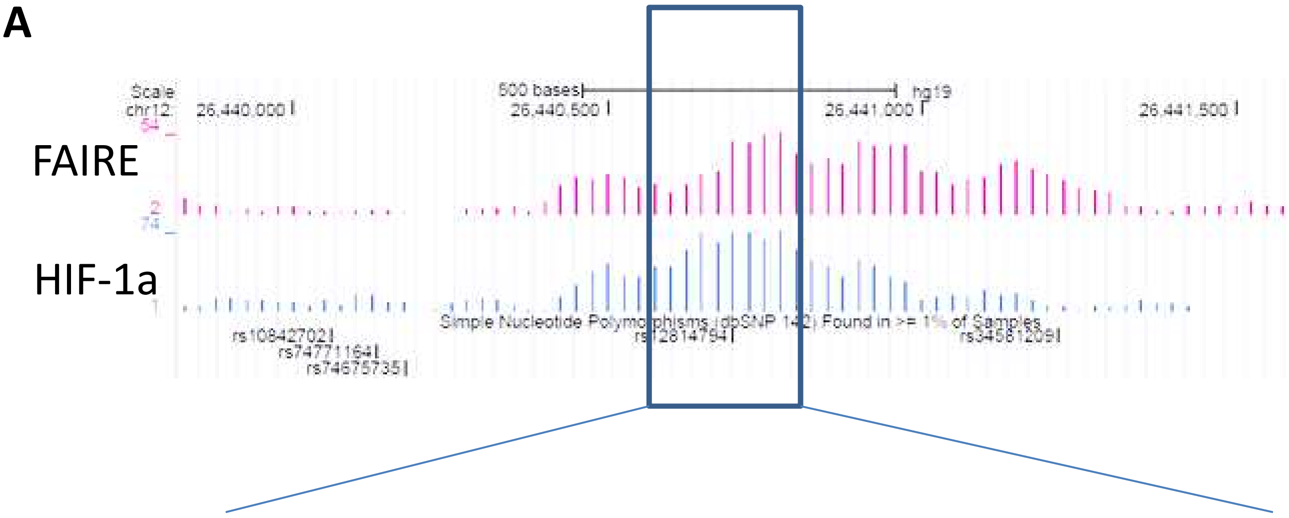

CCCTTCTGATTCAGCTGACCACTGTGCACTGTAGCGACGTGCAAGGGGTAGGAGTGGACACATTCCAGCTCGCTG

rs12814794

A>G

B

Sequence of the HIF-binding enhancer

TTGGAACCACAGCTCCTCCATGGCCAAATTTCTCTTTATGTCTCAGGTTT
CAGAGCATGGCCTAATATTTCCCGTACAGACAGGCCGCTCCGCTGCCCTT
CTGATTCAGCTGACCACTGTGCACTGTAGCGACGTGCAAGGGGTAGGAGT
GGACACATTCCAGCTCGCTGGGGTTTGGAACGGCACCTCTCATTCCCAGA
GAAGGGATTCTCTGTGGAGGCTGAGTTGGCAGTGCTCAAGCTTGCCTTGC
GGTTGGCACGTAGGCCATCAGCAATCATCTGAGTATCAACCTGCCAAGCT
GAGGCCGTCCTGGGGCACAGAGATTTATTGCTAACAGAGGAAAGAAAAGA
