# Supplementary material for: Multiple renal cancer susceptibility polymorphisms modulate the HIF pathway
Source: PLoS Genet. 2017 Jul 17;13(7):e1006872. doi: 10.1371/journal.pgen.1006872 (PMC5536434; doi:10.1371/journal.pgen.1006872)
Supplement: S1 Table — (PDF) [file pgen.1006872.s009.pdf]

Primers and assays

chr 12p12.1

**Expression**	fw	rev
HPRT	GACCAGTCAACAGGGGACAT	AACACTTCGTGGGGTCCTTTTC
BHLHE41	GCATGAAACGAGACGACACC	CGCTCCCCATTCTGTAAAGC
EGLN3	GGCCATCAGCTTCCTCCTG	GGTGATGCAGCGACCATCA

**FAIRE/ChIP**	fw	rev
chr 12p12.1	AGGTTTCAGAGCATGGCCTA	GCTGGAATGTGTCCACTCCT
Control region	AGCAAAGCAGAATGGAAGGA	CCCCACTGTGGAGTTTGTCT

**Taqman assays**	Cat. No.	Supplier
rs12814794	# C__31384137_10	Life Technologies
rs1048155	# C___1921778_10	Life Technologies
